# Evolutionary Dynamics of West Nile Virus in the United States, 1999–2011: Phylogeny, Selection Pressure and Evolutionary Time-Scale Analysis

**DOI:** 10.1371/journal.pntd.0002245

**Published:** 2013-05-30

**Authors:** Germán Añez, Andriyan Grinev, Caren Chancey, Christopher Ball, Namita Akolkar, Kevin J. Land, Valerie Winkelman, Susan L. Stramer, Laura D. Kramer, Maria Rios

**Affiliations:** 1 Laboratory of Emerging Pathogens, DETTD/OBRR/CBER, US Food and Drug Administration, Bethesda, Maryland, United States of America; 2 Idaho Bureau of Laboratories, Boise, Idaho, United States of America; 3 Bonfils Blood Center, Denver, Colorado, United States of America; 4 Creative Testing Solutions, Tempe, Arizona, United States of America; 5 American Red Cross, Gaithersburg, Maryland, United States of America; 6 New York State Department of Health, Albany, New York, United States of America, and School of Public Health, State University of New York at Albany, Albany, New York, United States of America; Centers for Disease Control and Prevention, United States of America

## Abstract

West Nile virus (WNV), an arbovirus maintained in a bird-mosquito enzootic cycle, can infect other vertebrates including humans. WNV was first reported in the US in 1999 where, to date, three genotypes belonging to WNV lineage I have been described (NY99, WN02, SW/WN03). We report here the WNV sequences obtained from two birds, one mosquito, and 29 selected human samples acquired during the US epidemics from 2006–2011 and our examination of the evolutionary dynamics in the open-reading frame of WNV isolates reported from 1999–2011. Maximum-likelihood and Bayesian methods were used to perform the phylogenetic analyses and selection pressure analyses were conducted with the HyPhy package. Phylogenetic analysis identified human WNV isolates within the main WNV genotypes that have circulated in the US. Within genotype SW/WN03, we have identified a cluster with strains derived from blood donors and birds from Idaho and North Dakota collected during 2006–2007, termed here MW/WN06. Using different codon-based and branch-site selection models, we detected a number of codons subjected to positive pressure in WNV genes. The mean nucleotide substitution rate for WNV isolates obtained from humans was calculated to be 5.06×10^−4^ substitutions/site/year (s/s/y). The Bayesian skyline plot shows that after a period of high genetic variability following the introduction of WNV into the US, the WNV population appears to have reached genetic stability. The establishment of WNV in the US represents a unique opportunity to understand how an arbovirus adapts and evolves in a naïve environment. We describe a novel, well-supported cluster of WNV formed by strains collected from humans and birds from Idaho and North Dakota. Adequate genetic surveillance is essential to public health since new mutants could potentially affect viral pathogenesis, decrease performance of diagnostic assays, and negatively impact the efficacy of vaccines and the development of specific therapies.

## Author Summary

West Nile Virus (WNV) is a mosquito-borne virus of African origin that is widespread around the world. The WNV life-cycle involves mosquitoes and birds, but humans and other animals can be infected, although they are not considered to be important players in the transmission cycle. Clinically, most WNV infections are unapparent, but the virus can disseminate to the central nervous system causing a potentially fatal neurological disease, especially in susceptible populations including elderly and immunocompromised individuals. West Nile virus can also be transmitted by organ transplant and by transfusion of blood and blood components. Like other arboviruses, WNV has the extraordinary capacity of growing in the different microenvironments represented by the invertebrate vector and the vertebrate hosts. From an evolutionary standpoint, the arrival of WNV in the US in 1999 represents a unique opportunity to explore the processes involved in the adaptation and dissemination of an arbovirus in a naïve environment. From the study of WNV sequences, we can not only learn about the evolutionary mechanisms that govern arboviruses, but also update diagnostic tests that rely on the detection of the viral genome upon the occurrence of mutations and study the existence of genetic markers that may be responsible for increases in clinical cases and their severity.

## Introduction

West Nile virus (WNV; genus *Flavivirus*, family *Flaviviridae*) is a mosquito-borne virus that is maintained in a bird-mosquito enzootic cycle, and is considered the most widely distributed flavivirus in the world [1], [2]. WNV can infect a broad range of vertebrate species including horses and humans which are considered dead-end hosts [3]. Most human infections (∼80%) are asymptomatic, and symptomatic infections vary from mild flu-like illness (∼20%) to fatal neuroinvasive disease (∼1%) [4]. WNV is estimated to have infected ∼4 million humans in the United States (US) between 1999 and 2011, causing over 31,000 serious illnesses, including 13,243 neuroinvasive disease cases and 1,261 deaths reported to the Centers for Disease Control and Prevention (CDC) (http://www.cdc.gov/ncidod/dvbid/westnile).

Historically, two major genetic lineages of WNV have been reported: lineages I and II [5], [6]. New WNV lineages have been proposed based on phylogenetic analysis of complete or partial genomes of new isolates, and the virus has been postulated to have up to five distinct lineages [7]–[9]. Clade 1a of lineage I contains isolates from Africa, Europe, the Middle East, Russia, and the Americas, and includes all recent isolates from US outbreaks [10]. In recent years, recurrence of the transmission of WNV to humans in Europe has intensified, where strains from both lineage I and II have been reported to be in circulation, and where lineage II WNV has been linked for the first time to neuroinvasive disease [11]–[14].

During 1999, the first cases of WNV in the Americas were reported in New York City. Analysis of WNV sequences from human cases from the 1999 epidemic revealed that these strains belong to WNV lineage I, and that US genotype was subsequently named genotype NY99. The strains from the WNV NY99 genotype have been considered to have a Middle Eastern origin because of their close relationships to a strain isolated from Israel in 1998 (IS-98 STD) [15]. Subsequently, extensive phylogenetic analysis has suggested that both the US and Israeli WNV strains have an African origin [10].

In 2001, a new genotype (termed WN02) emerged in the US becoming increasingly prevalent in 2002, and eventually displacing the ancestor genotype NY99 [16], [17]. The WN02 genotype is characterized by 13 conserved silent nucleotide mutations including 1 amino acid (aa) substitution (V_159_A) in the envelope protein (E) gene [16]. The new genotype became dominant in the Americas presumably due to its ability to disseminate more efficiently in domestic *Culex pipiens* and *Culex tarsalis* mosquitoes as compared to the NY99 genotype [18], [19].

The genomic RNA of WNV is approximately 11 kb in length, and contains 10 genes within a single open reading frame (ORF) that encodes for a single polyprotein, flanked by 5′ and 3′ untranslated regions (UTR). The approximately 3,400 aa WNV polyprotein is processed by cellular proteases and by the viral NS2B-NS3 protease into 3 structural (C-prM-E) and 7 non-structural proteins (NS1, NS2A, NS2B, NS3, NS4A, NS4B and NS5) [20]. Genetic variation in flaviviruses can occur via single base mutations, small insertions and deletions within the linear evolutionary pathway of the virus lineage, and more rarely by recombination events [21]. Viral adaptation through fixation of spontaneous mutations is considered an important factor potentially associated with recurrence of WNV outbreaks in the New World [22].

In this study, we examined the genetic variation and evolutionary processes acting upon WNV strains sampled in the US from different hosts including birds, mosquitoes and humans, and performed comparisons on the phylogeny and natural selection pressure using complete sequences from the US available in the GenBank database. We report here a new cluster termed MW/WN06, positioned within the recently described genotype SW/WN03, which consists of isolates obtained from human and bird specimens collected from Idaho and North Dakota in 2006–2007. Persistence of the transmission of strains from cluster MW/WN06 in the Midwest region of the US, as well as phenotypic characteristics such as virulence and dissemination capacity of those strains needs to be further studied.

## Methods

### Ethics statement

All human specimens used in this study originated from blood donors who signed the blood center's Institutional Review Board (IRB) approved informed consent and tested reactive in nucleic acid assays used to screen donations for WNV RNA. Prior to shipment, these specimens were anonymized (unlinked). Use of these already-existing unlinked specimens has been approved as exempt by the US Food and Drug Administration (FDA) IRB (Human Subjects Research - Exempt RIHSC Protocol #127B).

### Samples

A total of 32 WNV isolates were sequenced and included in this study. Twenty-nine (29) were obtained from human plasma samples derived from blood donors who tested reactive for WNV RNA by FDA-approved commercial nucleic acid test assays used to screen blood donations. These specimens were randomly selected from our repository and cover 12 states of the continental US: Arizona (AZ), California (CA), Colorado (CO), Idaho (ID), Louisiana (LA), North Dakota (ND), Nevada (NV), New York (NY), Mississippi (MS), South Dakota (SD), Texas (TX) and Utah (UT), spanning from 2006–2011. Of the remaining three isolates included here, two were from avian specimens from ID and one from a mosquito pool from NY. These specimens were positive for WNV by RT-PCR performed at their respective state department of health laboratories and were provided to us as field specimens for genetic studies. All isolates had the complete open reading frame sequenced and were included for analysis (Table 1 and Table S1).

**Table 1:** List of WNV isolates completely sequenced in this study.

	Isolate ID	Host	Collection year	Location	GenBank no.
1	NY10-03	Mosquito	2003	NY	JQ700437
2	ID21bird-07	Avian	2007	ID	JF957171
3	ID28bird-07	Avian	2007	ID	JF957172
4	ARC10-06	Human	2006	ID	JF957161
5	ARC13-06	Human	2006	ID	JF957162
6	ARC17-06	Human	2006	ID	JF957163
7	ARC23-06	Human	2006	ID	JF957164
8	ARC27-06	Human	2006	ID	JF957165
9	ARC33-06	Human	2006	UT	JF957166
10	BSL106-06	Human	2006	ND	JF957167
11	ARC140-07	Human	2007	ID	JF957168
12	CO4-07	Human	2007	CO	JF957169
13	CO5-07	Human	2007	CO	JF957170
14	BSL173-08	Human	2008	AZ	JF957173
15	BSL176-08	Human	2008	NV	JF957174
16	BSL2-09	Human	2009	NV	JF957175
17	BSL5-09	Human	2009	AZ	JF957176
18	BSL6-09	Human	2009	NV	JF957177
19	BSL11-09	Human	2009	NV	JF957178
20	BSL18-09	Human	2009	LA	JF957179
21	BSL20-09	Human	2009	NV	JF957180
22	BSL22-09	Human	2009	SD	JF957181
23	BSL24-09	Human	2009	TX	JF957182
24	BSL27-09	Human	2009	TX	JF957183
25	CO7-09	Human	2009	CO	JF957184
26	BSL2-10	Human	2010	AZ	JF957185
27	BSL3-10	Human	2010	AZ	JF957186
28	BSL4-11	Human	2011	AZ	JQ700438
29	BSL6-11	Human	2011	MS	JQ700439
30	BSL23-11	Human	2011	AZ	JQ700440
31	BSL24-11	Human	2011	CA	JQ700441
32	BSL26-11	Human	2011	NY	JQ700442

### Viral isolation, RNA extraction and Reverse Transcription-Polymerase Chain Reaction (RT-PCR)

Virus isolation was performed in African green monkey kidney (Vero) cells (ATCC # CCL-81) as described previously by Grinev et al. [22]. A single Vero cell passage was performed to expand the virus in order to obtain the required RNA concentration for sequencing purposes. Cell culture supernatants were harvested when extensive cytopathic effect was observed, clarified by centrifugation to remove cell debris and frozen at −80°C until further analysis. Cell culture supernatants (140 µl) were subjected to RNA extraction using the QIAamp viral RNA extraction kit (Qiagen, Valencia, CA) according to the manufacturer's protocol. Extracted RNA was stored at −80°C until further analysis. Reverse transcription reactions and PCR amplification were performed as described previously [22].

### DNA sequencing

After agarose gel electrophoresis, PCR products covering the entire WNV genome were purified using the MinElute Gel Extraction Kit (Qiagen) according to the manufacturer's instructions, and both strands were subjected to direct Sanger sequencing using the amplification primers and additional internal sequencing primers, with a minimum of 4X coverage. Sequencing reactions were performed as described elsewhere [22]. Amplification and sequencing primer sequences are available upon request from the authors. Nucleotide sequences reported in this paper are available in the GenBank database (accession numbers JF957161–JF957186 and JQ700437–JQ700442).

### Phylogenetic analysis

For the phylogenetic analysis, in addition to our newly sequenced WNV strains from human origin (Hu-WNV), n = 29, a search for fully sequenced WNV from the US in the GenBank database was performed. All the WNV ORF (10,299 nucleotides, nt) sequences available in the GenBank database as of January 2012 (∼400 sequences) were retrieved and analyzed for the presence of identical sequences that were subsequently removed to avoid duplications in the successive analyses. Sequences known to be laboratory strains (adapted to grow in either animals and/or cell culture) and therefore subjected to artificial selection, as well as sequences bearing at least one ambiguous nt reported in the ORF of the virus were also excluded from the analyses. Additionally, two WNV sequences of avian origin from Mexico which were shown to be related to US strains were also included in the dataset. The final dataset comprises a total of 363 WNV ORF sequences constituted from strains derived from various hosts including birds (n = 133), mammals (humans, n = 32; and a single sequence each from horse and squirrel specimens) and mosquitoes (n = 167) available in the GenBank, in addition to the newly sequenced strains produced in our laboratory from human (n = 29) and avian specimens (n = 2). For a complete list of strain names, host, state of origin and GenBank accession numbers, see Table S1.

Maximum likelihood (ML) and Bayesian approaches (B) were used to generate phylogenetic trees, using parental strain IS-98 STD (AF481864) as an outgroup to root the trees. The selected strains were aligned using MUSCLE implemented in MEGA5 [23] and the ML analyses were conducted in PhyML [24]. Bayesian information criterion (BIC) was used to determine the model of nt substitution that best fit the data using the selection tool available in MEGA5. The model that best fit the data was the General Time Reversible (GTR) + Γ + I model. For the analyses performed in PhyML, the initial tree was generated by BIONJ with tree improvement using Nearest Neighbor Interchange (NNI) and Subtree Pruning and Regrafting (SPR) methods and optimization of both topology and branch lengths. Branch support was done by the approximate likelihood ratio test (aLRT) and non-parametric branch support based on a Shimodaira-Hasegawa-like (SH-like) procedure [24], [25], as implemented in the on-line version of PhyML (http://www.atgc-montpellier.fr/phyml).

Furthermore, the Bayesian inference method implemented in the program MrBayes v3.1.2 [26] was used to analyze the WNV dataset. For the substitution model, the General Time Reversible (GTR) + Γ + I model was determined to be the best fitted for the data based on Aikake information criterion scores calculated by jModelTest 0.1.1 [27], and used with successive branch swapping. Four Markov Chain Monte Carlo (MCMC) chains were run for 10,000,000 generations, sampling every 100 generations, with the first 10,000 sampled trees discarded as burnin. Finally, a 50% majority rule consensus tree was constructed from the posterior distribution of trees.

### Selection pressure analysis

We performed a comprehensive selection analysis in the ORF of WNV strains isolated from all host species (ALL dataset) and from a dataset containing only Hu-WNV (H dataset). Prior to the selection analysis, recombination was assessed by the Recombination Detection Program v.4.13 [28] and no signals of recombination were detected. In addition to the ORF sequence datasets, the ALL and H datasets were further partitioned into each of the 10 individual WNV protein genes and analyzed for selection pressure. In order to analyze the natural selection mechanisms acting on the codons of the ORF and each of the 10 individual genes of WNV we used the HyPhy (Hypothesis testing using phylogenies) package under the Datamonkey web-server (www.datamonkey.org) [29]. The *dN/dS* ratios (ω) were calculated using three different codon-based maximum likelihood approaches (CBML): the single-likelihood ancestor (SLAC), fixed-effects likelihood (FEL) and the internal branch fixed-effects likelihood (IFEL) [30], [31]. Due to alignment size restrictions from the server, the random-effects likelihood method (REL) was only used to evaluate the H dataset.

The mixed effects model of evolution (MEME) method, a branch-site model, was also employed for studying the selection pressure in the different host datasets. This method is a generalization of FEL, which models variable ω across lineages at an individual site being able to detect smaller proportions of branches evolving subject to positive selection that would otherwise be detected as “negatively” selected by FEL [32], [33]. For all the methods employed for the ORF datasets, the GTR model was used as nt substitution bias model, while for the individual gene datasets the TN93 model was used. Trees were inferred by the neighbor-joining method and significance levels were set to p<0.1 or Bayes factor>50.

### Time-scale analysis

Evolutionary rates for the Hu-WNV sequences (H dataset, n = 61) were calculated by using the Bayesian MCMC approach employed by BEAST ver. 1.6.2 [34]. The data were analyzed using the TN93+Γ_4_ substitution model. We tested four parametric demographic models (constant population size, expansion, exponential and logistic growth) and the non-parametric Bayesian Skyline plot (BSP) model, under both strict and relaxed uncorrelated lognormal (UCLN) molecular clocks. Models were compared by calculating the Bayes Factors (BF), which are the ratio of the marginal likelihoods (marginal with respect to the prior) of the models compared. For each coalescent model we estimated the marginal likelihoods using the method described by Newton and Raftery [35] and modified by Suchard et al. [36], and evidence against the null model (model with the lower marginal likelihood) was determined as previously described [37]. Four MCMC chains were run until convergence to the stationary distribution was achieved for each demographic and clock model. Each independent chain was then combined with a burnin value set to 10% generations. The maximum clade credibility tree (MCC) was generated for each model. The 95% highest posterior density (95% HPD) intervals were obtained to ascertain the uncertainty in the parameter estimates.

## Results

### Phylogenetic analysis

**Figure 1:**
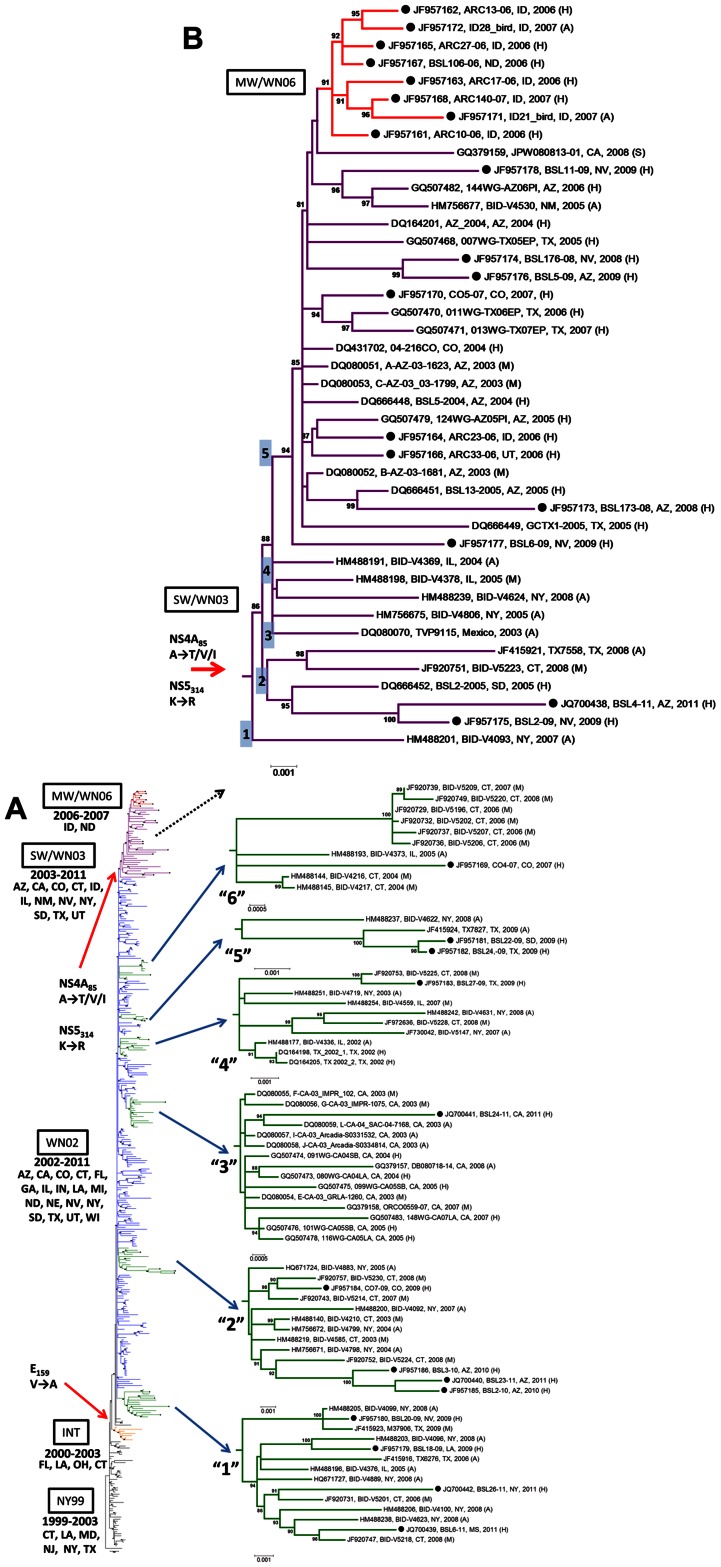
Consensus maximum-likelihood tree of all available WNV ORF from the US, 1999–2011 (n = 363). A) WNV genotypes are color-coded in the branches of the tree as NY99 (black), intermediate (orange), WN02 (blue), SW/WN03 (purple) and cluster MW/WN06 (red). States from which the analyzed strains were collected are shown below the label identifying the WNV genotypes. Nodes containing Hu-WNV sequences within genotype WN02 are shown highlighted in green and shown in detail. Amino acid changes defining important nodes are identified with red arrows. B) Detailed sub-tree showing genotype SW/WN03 and cluster MW/WN06. All Hu-WNV strains sequences derived from this study are highlighted with black circles (•). The numbers (1–5) on the nodes of the tree correspond to the SW/WN03 genotype groups as described by McMullen et al. (2011).

Phylogenetic analyses for WNV were performed by maximum-likelihood and Bayesian methods. These analyses included WNV sequences originating from avian, mosquito and human specimens, as well as one sequence each from horse and squirrel specimens available in the database (ORF, n = 363). The phylogenetic trees generated with this dataset revealed the presence of the clades (groups) already described during the study of the evolution of WNV in North America [10], [15], [16], [18], [20], [22], [38]–[43] (Figure 1A and Figure S5).

Phylogenetic analyses revealed that a total of 50 sequences clustered within the parental genotype NY99, which included strains collected from states located on the East and Gulf coasts, spanning from 1999–2003. The intermediate group, a cluster basal to the WN02 genotype [16], [18] included sequences from Florida (FL), LA and CT collected from 2000–2003. Strain TX04, reported previously to possibly be a recombinant strain of NY99 and WN02 genotypes [44], was also found located basal to the WN02 genotype.

Phylogenetic trees were constructed with the ALL dataset and color-coded according to year (1999–2011) and place of collection (US regions: Northeast, South, Midwest and West) and are available as Figures S1 and S2. The phylogenetic trees revealed that WNV sequences present a bush-like topology, i.e. in general, they are constituted by poorly differentiated clades with only a few clusters found to be geographically and/or temporally structured (Figure 1A). In addition, there is no clear host-origin (avian, mosquito, human, and other mammals) segregation of the strains in the constructed phylogenies (Figure S3). It is possible to note that most of the WNV sequences from the US available in the GenBank database originated from the states of NY, CT and IL, where tremendous efforts have been conducted to analyze the genetic composition of the WNV strains circulating there.

The genotype WN02 is constituted from samples collected from diverse regions of the US. Dispersed throughout the genotype WN02, we observed a number of WNV strains obtained from humans (Hu-WNV), including those that we are reporting here for the first time, grouping within several sub-clusters distributed across the phylogeny (Figure 1A). The Hu-WNV strains reported here are indicated in the text in italics and bold font. These Hu-WNV isolates located within genotype WN02 are positioned within six sub-clusters termed here clusters “1” to “6”. We found that a single Hu-WNV strain (***BSL24-11*** from CA) collected in 2011 was associated with the cluster containing the CA group (cluster D in the classification of Gray et al. [41]); termed here cluster “3”, which is constituted by strains sampled between 2003 and 2008. Thus this finding supports the notion that viruses from this cluster were still circulating in CA as late as 2011 (Figure 1A).

Previous analysis of the entire ORF of WNV isolates circulating in the Southwestern US (especially from TX) has shown that a new genotype had emerged in the region after 2003, termed genotype SW/WN03, for which five phylogenetic groups have been described before [43]. We have observed that some of the Hu-WNV isolates reported here clustered within the SW/WN03 genotype: ***BSL2-09*** and ***BSL6-09*** from NV in groups 2 and 4 (according to the classification in [43]), respectively, and ***BSL4-11*** from AZ in group 2. Group 5 of SW/WN03 was constituted by the largest number of strains within this newly described genotype, comprising strains from AZ, CA, CO, NM and TX and spanning from 2004–2008. We have identified 7 of the Hu-WNV strains sequenced here to cluster within group 5 of genotype SW/WN03: ***BSL176-08*** and ***BSL11-09*** from NV, ***BSL173-08*** and ***BSL5-09*** from AZ, ***CO5-07*** from CO, ***BSL33-06*** from UT and ***BSL23-06*** from ID (Figure 1B).

Of particular interest is the observation that five Hu-WNV strains from ID from 2006–2007 (***ARC10-06, ARC13-06, ARC17-06, ARC27-06, ARC140-07***), one Hu-WNV strain from ND from 2006 (***ARC106-06***), as well as two avian-WNV strains from ID collected in 2007 (***ID21_bird*** and ***ID28_bird***), clustered together and formed a distinct phylogenetic cluster within group 5 of SW/WN03, which has been termed here MW/WN06, after being described in the Midwestern US after 2006 (Figure 1B). Cluster MW/WN06 is particularly interesting from a phylogenetic perspective since it presents clear spatial and geographical structure, which is supported by high bootstrapping and Bayesian posterior probability values. This cluster is constituted by strains that were sequenced in an effort to study an ongoing epidemic in Midwestern states during 2006 and 2007, and included six human isolates and two bird isolates, thus representing viruses circulating in the competent host for that location.

Bayesian maximum clade credibility trees constructed with Hu-WNV from the US (n = 62) allow for the identification of the NY99, WN02, SW/WN03 genotypes and the clusters already recognized by the Maximum-likelihood analysis for the sequences reported here, i.e., clusters “1” to “6” in the WN02 genotype and cluster MW/WN06 in the SW/WN03 genotype, with high posterior probability values (Figure S4).

### Nucleotide changes and amino acid substitutions

When analyzing the nt and aa variation in the ORF of WNV for the whole set of 363 North American isolates, we found that overall, out of 10,299 nt, a total of 2,472 nt were polymorphic (24%), of which 1,186 (47.97%) were single polymorphisms, while 1,286 polymorphic sites (52.03%) were shared by 2 or more strains. Despite having 2,472 nt changes observed, only 492 of the 3,433 WNV-encoded aa (14.33%) were polymorphic, 331 of which were single polymorphisms (67.27%), leaving only 161 aa residues polymorphic for at least 2 of the 363 analyzed strains. As expected, most of the nt changes we found were silent transitions (U↔C, A↔G), accounting for ≈88% of the observed substitutions. Nucleotide mutations conserved in the studied Hu-WNV isolates compared to the complete genome of NY99 are shown in Table 2. There are between 9 and 25 nt differences among all human WNV isolates analyzed and the parental NY99 strain. All 29 Hu-WNV isolates completely sequenced here shared 5 nt mutations (T_1442_C, C_2466_T, A_4146_G, C_6138_T and T_8811_C), that are fixed in these Hu-WNV strains throughout 2006–2011 (Table 2). The substitution T_1442_C is the only non-synonymous mutation leading to the aa change E-V_449_A (V_159_A, in the E protein aa numeration). This substitution is present in all WNV strains sampled in the US since 2003, and therefore fixed in all members of the WN02 and SW/WN03 genotypes (Table 2).

**Table 2:** Nucleotide mutations present in studied human WNV isolates (2006–2011), compared to the prototype strain NY99.

		Gene																												
		prM	E				NS1	NS2A	NS3				NS4A			NS4B					NS5									# differences
Year	Strain/nt #	660	1320	1442	1974	2466	3399	4146	4803	6138	6238	6426	6721	6722	6765	6936	6996	7015	7209	7269	7938	8550	8620	8621	8811	9264	9352	9660	10062	
	NY99	C	A	T	C	C	T	A	C	C	C	C	G	C	T	T	C	T	A	T	T	C	A	A	T	T	C	C	T	
2006	ARC10-06	T	G	C	T	T	C	G	T	T	T	T	A	**•**	C	C	T	C	T	C	C	T	**•**	G	C	C	T	T	C	25
	ARC13-06	T	G	C	T	T	C	G	T	T	T	T	A	**•**	C	C	T	C	T	C	C	T	**•**	G	C	C	T	T	C	25
	ARC17-06	T	G	C	T	T	C	G	T	T	T	T	A	**•**	C	C	T	C	T	C	C	T	**•**	G	C	C	T	T	C	25
	ARC23-06	T	G	C	T	T	C	G	**•**	T	T	T	A	**•**	C	C	T	C	**•**	C	C	T	**•**	G	C	C	T	T	C	23
	ARC27-06	T	G	C	T	T	C	G	T	T	T	T	A	**•**	C	C	T	C	T	C	C	T	**•**	G	C	C	T	T	C	25
	ARC33-06	T	G	C	T	T	C	G	T	T	T	T	A	**•**	C	C	T	C	**•**	C	C	T	**•**	G	C	C	T	T	C	24
	BSL106-06	T	G	C	T	T	C	G	T	T	T	T	A	**•**	C	C	T	C	T	C	C	T	**•**	G	C	C	T	T	C	25
2007	ARC140-07	T	G	C	T	T	C	G	T	T	T	T	A	**•**	C	C	T	C	T	C	C	T	**•**	G	C	C	T	T	C	25
	CO4-07	T	**•**	C	**•** a	T	**•**	G	T	T	T	T	**•**	**•**	**•**	**•**	T	C	**•**	**•**	C	**•**	**•**	**•**	C	**•**	T	**•**	**•**	12
	CO5-07	T	G	C	T	T	C	G	T	T	T	T	A	**•**	C	C	T	C	**•**	C	C	T	**•**	G	C	C	T	T	C	24
2008	BSL173-08	T	G	C	T	T	C	G	T	T	T	T	A	T	C	C	T	C	**•**	C	C	T	**•**	G	C	C	T	T	C	25
	BSL176-08	T	G	C	T	T	C	G	T	T	T	T	A	**•**	C	C	T	C	T	C	C	T	**•**	G	C	C	T	T	C	25
2009	BSL2-09	T	**•**	C	**•** a	T	**•**	G	T	T	T	T	A	**•**	**•**	**•**	T	C	**•**	**•**	C	T	**•**	**•**	C	C	T	T	**•**	16
	BSL5-09	T	G	C	T	T	C	G	T	T	T	T	A	**•**	C	C	T	C	T	C	C	T	**•**	G	C	C	T	T	C	25
	BSL6-09	T	G	C	**•** a	T	C	G	T	T	T	T	A	**•**	C	**•**	T	C	**•**	C	C	T	**•**	G	C	C	T	T	C	22
	BSL11-09	T	G	C	T	T	C	G	T	T	T	T	A	**•**	C	C	T	C	T	C	C	T	**•**	G	C	C	T	T	C	25
	BSL18-09	**•** a	**•**	C	**•** a	T	**•**	G	T	T	**•**	T	**•**	**•**	**•**	**•**	T	C	**•**	**•**	C	**•**	**•**	**•**	C	**•**	T	**•**	**•**	10
	BSL20-09	**•** a	**•**	C	**•** a	T	**•**	G	T	T	**•**	T	**•**	**•**	**•**	**•**	T	C	**•**	**•**	C	**•**	**•**	**•**	C	**•**	T	**•**	**•**	10
	BSL22-09	T	**•**	C	**•** a	T	**•**	G	T	T	T	T	**•**	**•**	**•**	**•**	T	C	**•**	**•**	C	**•**	**•**	**•**	C	**•**	T	**•**	**•**	12
	BSL24-09	T	**•**	C	**•** a	T	**•**	G	T	T	T	T	**•**	**•**	**•**	**•**	T	C	**•**	**•**	C	**•**	G	**•**	C	**•**	T	**•**	**•**	13
	BSL27-09	T	**•**	C	**•** a	T	**•**	G	T	T	T	T	**•**	**•**	**•**	**•**	T	C	**•**	**•**	C	**•**	**•**	**•**	C	**•**	T	**•**	**•**	12
	CO7-09	**•** a	**•**	C	**•** a	T	**•**	G	T	T	**•**	T	**•**	**•**	**•**	**•**	T	C	**•**	**•**	C	**•**	**•**	**•**	C	**•**	T	**•**	**•**	10
2010	BSL2-10	**•** a	**•**	C	**•** a	T	**•**	G	T	T	**•**	T	**•**	**•**	**•**	**•**	T	C	**•**	**•**	C	**•**	**•**	G	C	**•**	T	**•**	**•**	11
	BSL3-10	**•** a	**•**	C	**•** a	T	**•**	G	T	T	**•**	T	**•**	**•**	**•**	**•**	T	C	**•**	**•**	**•**	**•**	**•**	**•**	C	**•**	T	**•**	**•**	9
2011	BSL4-11	T	**•**	C	**•** a	T	**•**	G	T	T	T	T	A	**•**	**•**	**•**	T	C	**•**	**•**	C	T	**•**	**•**	C	C	T	T	**•**	16
	BSL6-11	**•** a	**•**	C	**•** a	T	**•**	G	T	T	**•**	T	**•**	**•**	**•**	**•**	T	C	**•**	**•**	C	**•**	**•**	**•**	C	**•**	T	**•**	**•**	10
	BSL23-11	**•** a	**•**	C	**•** a	T	**•**	G	T	T	**•**	T	**•**	**•**	**•**	**•**	T	C	**•**	C	C	**•**	**•**	G	C	**•**	T	**•**	**•**	12
	BSL24-11	T	**•**	C	**•** a	T	**•**	G	T	T	**•**	**•**	**•**	**•**	**•**	**•**	**•**	C	**•**	**•**	C	T	**•**	**•**	C	**•**	**•**	**•**	**•**	9
	BSL26-11	**•** a	**•**	C	**•** a	T	**•**	G	T	T	**•**	T	A	**•**	**•**	**•**	**•**	**•**	**•**	**•**	C	**•**	•	**•**	C	**•**	T	**•**	**•**	10
	AA substitutions (polyprotein aa #, protein aa #)			V_449_A V_159_A									A_2209_T/I A_85_T/I										K_2842_R/E K_314_R/E							

Sequences were compared to the complete genome of WNV NY99 (AF196835).

a Nucleotide change considered “fixed” in the WN02 genotype found containing the NY99 genotype nucleotide sequence.

Surprisingly, nt changes from the parental genotype NY99 thought to be fixed and therefore supposed to be present in all WNV circulating the US after the emergence of genotype WN02, were found to be present in recent isolates reported here, i.e. C_660_T in the prM gene which was found as C_660_ in 8 Hu-WNV strains from 2009–2011, while C_1974_T in the E gene was found as C_1974_ in most of the analyzed samples collected after 2007 (present in 16 of the 29 Hu-WNV isolates reported here), prompting us to speculate that these sites reverted back to the parental genotype NY99 or represent strains that continue to circulate and that retain vestigial characteristics of the NY99 genotype despite the presence of genetic features considered distinctive of the WN02 genotype (i.e. the presence of T_1442_C, with the subsequent aa change V_449_A in E) (Table 2).

In the sequenced Hu-WNV, the number of deduced aa substitutions ranged from 4 to 13 when compared with NY99, most of which were conservative changes. In addition to the aa substitution E-V_159_A in the Envelope protein common to all WN02 genotype viruses, 17 Hu-WNV isolates from 2006–2011 shared the substitution NS4A-A_85_T. Interestingly, the aa change NS4A_85_ A→T is found in all sequences clustering in the SW/WN03 genotype, except for strains ***BSL13-05*** and ***BSL173-08***, which have an A→I substitution. Sixteen (16) of 29 Hu-WNV isolates reported here shared the substitution NS5-K_314_R, while for one strain (***BSL24-09***) the non-conservative NS5-K_314_E was identified (Table 2).

### Selection pressure analysis

The *dN/dS* ratios (ω) for the ALL and H datasets were 0.105 and 0.127, respectively, suggesting that WNV is subjected to strong purifying (negative) selection, as has been previously observed for other flaviviruses like Dengue virus [45], [46] (Table 3). For the ALL dataset, which contains 363 strains isolated from mosquito, avian or mammalian hosts, we found evidence, supported by at least 3 methods, of positive selection in 5 codons in the WNV ORF (938-NS1_147_, 1841-NS3_336_, 2209-NS4A_85_ and 2842-NS5_314_), while only 2 codons (1841 and 2209), were detected as having been positively selected for the Hu-WNV dataset (H dataset) under the same stringent analysis conditions (Table 3). Additional, more inclusive analyses of selection pressure were conducted including the recently developed MEME model (a branch-site method), for which results suggest that a larger number of sites in the WNV genome may be subjected to positive pressure and may have been evolving under episodic directional selection [32] (Tables S2–S4).

**Table 3:** Selection pressure analysis of WNV strains collected in the US (1999–2011), by host dataset.

Dataset^a^	Codon	Protein and AA #	Methods^c^				
			FEL IFEL SLAC MEME				REL
			*P* value				BF
H (n = 61), ω^b^: 0.127 231 negatively selected sites							
1	1841	NS3-L_336_S	0.10	0.07	*0.17*	0.10	245
2	2209	NS4A-A_85_T/I	0.07	0.02	0.30	0.07	3,614
ALL (n = 363), ω^b^: 0.105 963 negatively selected sites							
1	938	NS1_147_	0.07	0.05	0.49	0.07	n.d.
2	1841	NS3_336_	0.09	0.07	*0.16*	0.09	
3	2209	NS4A-A_85_T	0.002	0.01	0.03	0.002	
4	2842	NS5_314_	0.04	0.02	0.08	0.01	

Presented codons were detected by the methods employed in HyPhy (Datamonkey server), in the viral Open Reading Frame (3,433 codons).

a H = Human, ALL = all hosts.

b ω = *dN/dS* ratio.

c FEL = Fixed effects likelihood, IFEL = Internal Fixed effects likelihood, SLAC = Single-likelihood ancestor counting, MEME = Mixed Effects Model of Evolution, REL = Random Effects likelihood, BF = Bayes factor, n.d. = not done. All codons present in the table are recognized by at least three methods. *p* values in italics represent codons detected to be under positive selection, not significantly, but close to *p* threshold (0.1).

In the analysis of selection pressure of the individual genes (gene-by-gene), for the ALL dataset, the structural protein genes (C, prM, E) and the non-structural protein NS2B were the only genes that did not reveal codons detected to be under positive selection by at least two of the employed methods. The remaining WNV non-structural protein genes had one or more sites detected under positive selection by at least two of the employed methods (Table S3). When selection pressure was analyzed gene-by-gene in the H dataset, one codon each for the E, NS1, NS3 and NS4A genes, and five sites in the NS5 gene were found to be subjected to positive selection by at least two of the methods employed in the Datamonkey server (Table S4). Codon 85 in NS4A was also found to be under positive selection when we performed a gene-by-gene selection pressure analysis of the H dataset using the Bayesian empirical method employed in the Selecton server [47], with the M8, beta + w> = 1 evolutionary model (data not shown).

Furthermore, we conducted an additional selection pressure analysis in datasets of WNV sequences segregated by avian and mosquito-host origin. For these datasets, a number of sites were also found to be subjected to purifying selection. In some occasions, positively detected sites were only found in one host-origin dataset but not in the other (e.g. a codon found positively selected with strong statistical support in the mosquito or avian dataset that was not identified in the rest of the datasets) (Table S2), which may be a signal of modest host-specific positive selection bias occurring for certain codons during the diversification of WNV in the US. Taken together, the results from our natural selection analysis for WNV in the US suggest that the number of positively selected sites detected with statistical significance varies depending upon the host origin and the number of sequences analyzed.

### Time-scale analysis

The evolutionary rates for WNV were determined for the H dataset (US human origin WNV strains + strain IS-98 included as an outgroup, n = 62), using both strict and relaxed molecular clocks, 4 parametric demographic models and the non-parametric BSP model. An attempt to perform a similar time-scale analysis for the ALL dataset failed to converge after more than 4*×*10^8^ generations, which as has been noted before, seems to be due to computational constraints [41].

Results for the evolutionary time-scale analysis for the H dataset are summarized in Table S5. To assess the population dynamics for this dataset, we compared results on the parametric and the BSP models, where the BSP with the relaxed molecular clock (UCLN) was found to be the best-fitted model based on results on the BF comparison of the marginal likelihoods for the models assessed. Under this model, we calculated the mean nucleotide substitution rate (MNSR) to be 5.06*×*10^−4^ substitutions/site/year (s/s/y), 95% HPD = 4.44–5.70*×*10^−4^ s/s/y). The time to most recent common ancestor (*tMRCA*) for the whole dataset was 15.57 years ago (95% HPD = 14.23–16.98 years) or around 1995. Since the analysis included the parental strain from Israel (IS-98 STD) as outgroup, the calculated year (1995) corresponds to the *tMRCA* for that strain (Table S5). In the case of US Hu-WNV strains (including all genotypes), the mean *tMRCA* calculated was of 13.64 years ago (95% HPD = 12.77–14.64 years ago) or around 1997, which is between 1–2 years before the virus was identified in the US, more specifically in New York City during 1999.

**Figure 2:**
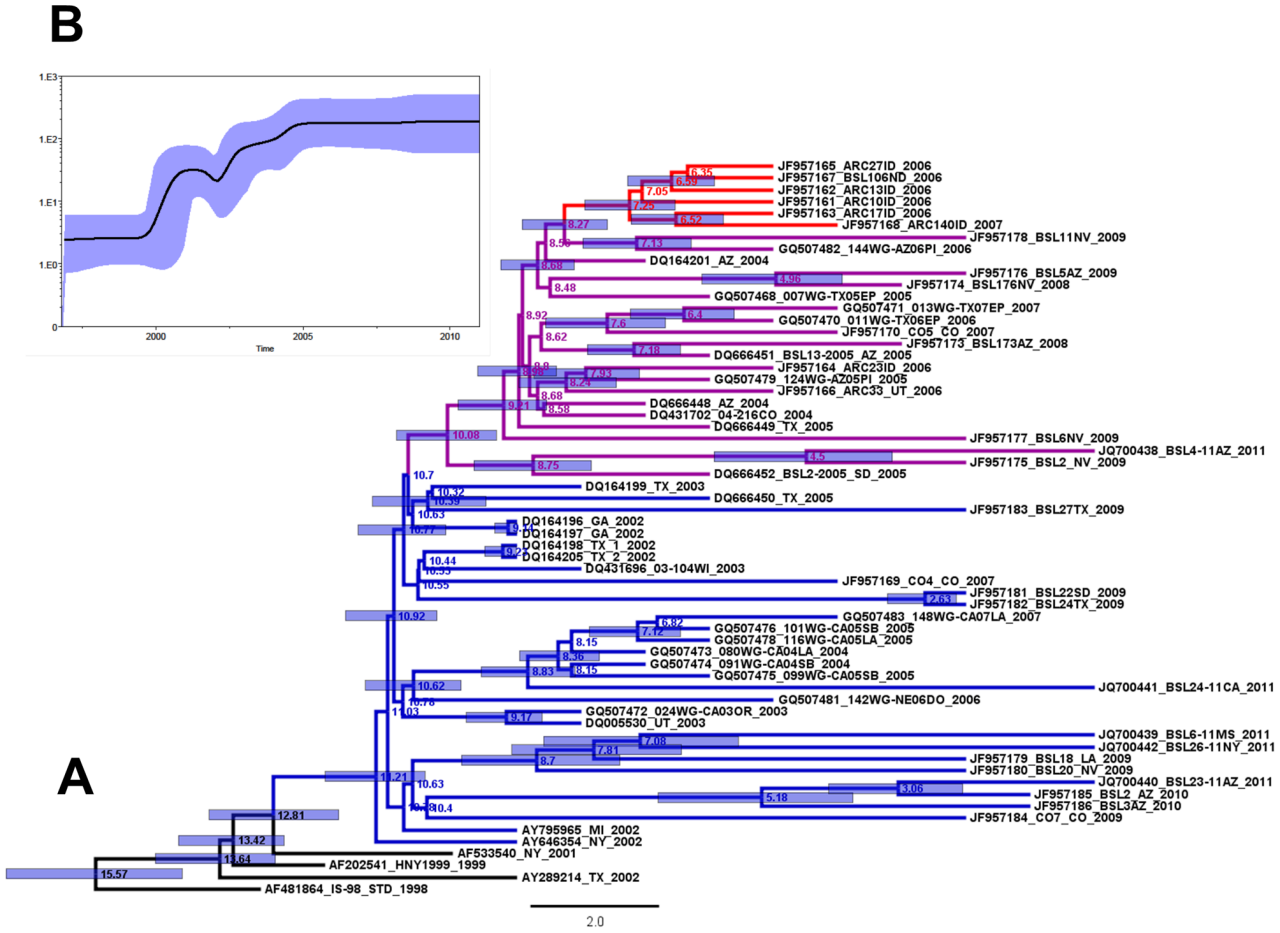
Maximum clade credibility tree from Bayesian analysis of US human-origin WNV strains, 1999–2011. A) WNV genotypes are color-coded in the branches of the tree as NY99 (black), WN02 (blue), SW/WN03 (purple) and cluster MW/WN06 (red). The mean time to the most recent common ancestor (*t_MRCA_*) is shown in each principal node. The 95% highest probability density (95% HPD) for each node age, are shown as blue bars. B) Bayesian coalescent inference of genetic diversity and population dynamics using the Bayesian Skyline plot available in BEAST 1.6.2., for US WNV of human origin (1999–2011). X axis represents years of study and y axis the relative genetic diversity product of the effective population size. Black line represents the mean estimate and the blue shadow, the 95% HPD.

A maximum clade credibility tree (MCC) derived from the estimations obtained with the best models (BSP and UCLN) was constructed and the age for each node is shown (Figure 2A). Similar topology is observed in the MCC tree in comparison to the maximum-likelihood and Bayesian consensus phylogenetic trees constructed with the H dataset (data not shown). The mean age for the WN02 genotype was calculated to be about 11 years (or around 2000), which suggests that after the appearance of this WNV genotype, *in situ* evolution has occurred independently in several places. For the SW/WN03 genotype, the mean node age is about 10 years (2001), and for the newly recognized cluster MW/WN06, is of approximately 8 years. The Bayesian skyline plot of Hu-WNV strains shows that after the identification of the virus in the US in 1999, a period of high genetic variability was observed until approximately 2002, which is congruent with the observations of Snapinn et al. [17]. This high variability period (period 1) was followed by a brief period of contraction, after which another steep period of high variability (period 2) is observed until around 2005, where the genetic diversity of the WNV population infecting humans appears to have reached a stability point (Figure 2B).

## Discussion

Since 1999, WNV has spread from New York City throughout the US and the Americas including Canada, Mexico, the Caribbean, and more recently, South America [3]. Previous studies have examined the evolutionary dynamics and spread of WNV after its introduction in North America, which has been described as a unique scenario to study the invasion and adaptation mechanisms of a pathogen, more specifically a flavivirus, to a naïve environment [10], [15], [16], [18], [20], [22], [38]–[43].

This study focuses on the evolutionary processes (phylogeny, selection pressure and evolutionary time-scale analysis) affecting WNV strains circulating in the US since its identification in the country in 1999 until the 13^th^ consecutive epidemic in 2011, through the study of entire viral genome sequences (ORF), with special emphasis on the study of sequences obtained from viremic humans. After its arrival in the US, the parental WNV genotype NY99 has evolved *in situ*, and starting in 2001, the new genotype WN02 emerged and was reported to have completely displaced genotype NY99 by 2003, after widespread distribution across North America [22], [43]. The bush-like topology of WNV phylogenies suggests that the virus in the US has undergone population expansion after a single viral introduction. This phenomenon can be explained by the sudden pressure exerted by ecological factors, i.e. the vector and host species, to which the virus had to adapt in the US. The existence of *in situ* evolution of WNV in different regions of the US has been demonstrated by results obtained by us and others, and positive selection in sites conferring increased viral fitness seems to have occurred. Positively selected codons have been analyzed for their involvement in generation of lineages, and this information can be found accompanying the phylogenetic trees reported here (Figure 1). Although temporal and geographical structure is clearly evident in comprehensive sampled WNV phylogenetic reconstructions, several studies have reported a lack of geographic structure in the US based on phylogenetic analyses using prM and E gene sequences [37]–[40], [48]. Analyses using entire WNV genome sequences have shown a better resolution of the geographical structure of the strains than that obtained from partial genomic sequence analysis [22], [42]–[44]. However, the non-structural proteins NS3 and NS5 genes have shown to provide phylogenetic reconstructions close to those obtained when entire genomes or ORF sequences are used [41].

Our comprehensive phylogenetic analysis of WNV isolates from the US demonstrates that with few exceptions, WNV strains from the WN02 genotype circulating in the country are poorly differentiated spatial and temporally, and these results correlate with other recently published studies on WNV phylogeny in the US [10], [41], [43]. The WN02 genotype differs from the NY99 genotype by only 1 aa substitution (E-V_159_A) and 13 silent nt mutations [16]. We have found that two other aa substitutions (NS4A-A_85_T and NS5-K_314_R) appear to have become fixed for genotype WN02, and consequently are also fixed in the newly reported genotype SW/WN03. Positive selection of these two aa substitutions potentially could impact viral fitness, phenotype and virulence.

WNV genetic variation in the US has been postulated to have occurred in very defined geographical areas (“niches of evolution”), in which the variant viral strains accumulate genetic changes while adapting to the local ecological conditions and may either perpetuate in that area; where it could be disseminated to other regions by migrating birds or other less understood mechanisms, or become extinguished if sustained transmission of such strains is not maintained [49], [50]. Studies on the phylogeography of WNV in the US [50] have shown a westward dissemination of WNV lineages that matches the observed spatio-temporal incidence of the virus and that some of the viral lineages exhibit atypically rapid and long-distance travel. These authors reported that the WNV epidemic in the US cannot be adequately described by homogeneous dispersal, and instead reported that it has been critically shaped by high variation in dissemination of infected hosts.

We have identified and report here for the first time, a cluster of WNV clearly defined spatially and temporally that grouped within the genotype SW/WN03 and that is constituted by isolates from human and birds circulating in the states of ID and ND during 2006 and 2007. We have termed this group as cluster MW/WN06 for the location of these states within the Midwest of the US. The detected local concentration of closely related isolates in states of the Midwestern US is likely due to initial introduction of one or few genetically similar viral strains in the area with rapid spread to mosquitoes and local birds, amplifying the initially carried genome and thus human infections in that area would reflect the colonizing genotype. Other places in the US for which *in situ* evolution and dissemination have been reported include Texas and California [43], [51].The virulence of some of these lineages has been studied and results indicate that localized selection for attenuated strains may occur and that they may later become extinguished [16], [51], [52]. The virulence of the MW/WN06 cluster is currently unknown. Molecular epidemiology and virulence studies are ongoing in order to determine the biological characteristics of this genotype that appears to have emerged *in situ* and may have the potential to expand further.

Analysis of WNV isolates sequenced in our study as well as reports by others [22], [43] indicate that although most new nt mutations detected year-to-year are not fixed, WNV continues to diverge in the US. The data showed that further genetic drift has occurred in the US since our report in 2008 analyzing WNV sequences collected from humans between 2002 and 2005 [22]. A time-scale analysis of WNV was performed on sequences of the entire ORF in WNV strains isolated from infected humans, mostly from viremic blood donations collected throughout the US. For this group of sequences we found a MNSR of 5*×*10^−4^ s/s/y, which is similar to what has been reported by May et al. [10], where the MNSR among all isolates analyzed (including sequences collected from various hosts and from all WNV genotypes collected worldwide) was of 7.55*×*10^−4^ s/s/y, although substitution rates were found by these authors to vary when the phylogenetic groups are compared to the others, ranging from 2.24*×*10^−4^ to 1.06*×*10^−3^ s/s/y. Other MNSR calculations for WNV have been performed using either E [17], [37], [39], [41] or the non-structural proteins NS3 and NS5 gene sequences [41], mainly derived from WNV sequences from mosquito and/or avian specimens collected in the US. These studies revealed a broad range of MNSR from 3*×*10^−4^ to 8*×*10^−3^ s/s/y depending upon the host population, gene and coalescent parameters (demographic and clock models) employed for the calculations. However, when concatenated NS3 and NS5 protein genes were used to calculate evolutionary rates and *tMRCA*, the analyses failed to converge after 3*×*10^8^ generations, suggesting that current computational resources are insufficient for large alignments [41].

RNA viruses exist in nature as “*quasispecies*”, or more accurately as mixtures of closely related but genetically diverse populations upon which selection acts, and such degree of variation derives from the relatively high replication rates, population size, and error rates that occur during replication of their genomes by their error-prone polymerases. The degree of diversity and the potential for evolution within such a population at any given time is a product of the balance between selection (positive or negative pressures which impact the relative fitness of variants) and genetic drift (the accumulation of random mutations during replication) [53]. Despite the fact that numerous nt changes have been reported during the course of WNV evolution in North America, negative selection appears to constrain changes at the protein level. A number of studies have shown only a low level of positive selection in WNV isolates from North America [20], [22], [39], [42]. The low level of positive selection suggests that most aa changes in WNV in North America have been the result of genetic drift.

We analyzed the selection pressure acting upon codons of the WNV isolates collected in the US using codon-based methods (FEL, IFEL, REL and SLAC), in addition to a recently developed branch-site method (MEME). The MEME method is now recommended over the traditional maximum-likelihood codon-based methods since it appears to be superior to other methods for identifying both episodic and pervasive positive selection [33], which may have led in the past to underestimation of the number of positively-selected sites in the studied WNV datasets. This warrants further studies to analyze the biological significance of these findings. There is evidence in favor of increased genetic diversity in mosquitoes when compared to birds [37], [38]. We have observed indication of host-dependent selection pressure when we conducted a separated selection analysis in host specific datasets of WNV sequences (i.e. avian, mosquito, human and other mammals). We speculate that this evidence supports the existence of positive selection bias within different WNV hosts in the US, and thus the study of such host-dependent selection constraints warrants further investigation.

Two aa residues (NS4A_85_ and NS5_314_) found to be subjected to positive selection in the ALL dataset were mapped to the phylogeny and found to be involved in the formation of the SW/WN03 genotype, while two other residues, E_431_ and NS2A_224_ were found in the California cluster within WN02, and in a number of NY99 and SW/WN03 genotype isolates, respectively. Sites NS4A_85_ and NS5_314_ were found to be under positive pressure by both groups ([43] and our results). Armstrong et al. [44] reported positive selection identified by FEL and SLAC at NS4A_135_, strong negative selection in E_159_, and convergent or parallel evolution across viral lineages by mapping aa substitutions to the WNV phylogeny, in which it was noted that the same substitutions occasionally occurred independently in different lineages. In that study, positive selection at position NS4A-V_135_M was found to be present in sequences from CT and TX, and it was speculated that this change has the potential to alter RNA replication and interferon evasion mechanisms [44]. The observed positive selection at site NS4A-A_85_T, found in the analyzed datasets in our study and by others [43], may also have the potential to affect putative functions of the NS4A protein. In addition, our analyses show that a number of codons in the NS5 protein are subjected to positive pressure (Tables S2, S3, S4). NS5 is the viral RNA- dependent RNA polymerase; an enzyme that exhibits extraordinary flexibility since it is subjected to very different biochemical conditions while in either the arthropod vector or the bird and mammal hosts. Single amino acid changes in NS5 have been found to have an impact for WNV replication in different hosts [54]. In this study, codon NS5_314_ was found to be subjected to strong positive selection, and the effects of the selection of this codon need to be studied in detail to elucidate its possible role for the replication of WNV in the different natural hosts.

Our findings of Hu-WNV clustering within every genotype of WNV and across the geography of the US, supports the notion that although humans are considered dead-end hosts for WNV and therefore thought not to play an important for the lifecycle of the virus, human infections by the virus continue to occur and represent an important risk for public health in general and for the blood supply of the country. The expansion of WNV across the US makes it necessary to analyze the genetic make-up of the virus in the different localities in which the virus circulates. Viral adaptation of WNV in mosquitoes and birds is considered to have played a major role in the spread of WNV in North America; thus additional studies are needed to further investigate phenotypic differences of these circulating variants of WNV *in vitro* and *in vivo* using mosquito and avian models. Furthermore, the establishment of WNV in the US represents a unique opportunity to understand how an arbovirus adapts to new hosts and spread in a naïve environment. Adequate WNV genetic epidemiological surveillance is also essential for public health since new mutants could potentially affect viral pathogenesis, decrease performance of diagnostic and blood/organs screening assays, and negatively impact the efficacy of vaccines and the development of specific therapies.

## Supporting Information

Figure S1 Consensus maximum-likelihood tree of WNV ORF, from the US (1999–2011), by year of collection (n = 366). Strain IS-98 STD is used as outgroup to root the tree. Year of collection is color-coded according to the insert at the bottom of the figure. Taxon names at the tip of the branches correspond to GenBank accession codes.
(TIF)

Figure S2 Consensus maximum-likelihood tree of WNV ORF, from the US (1999–2011), by place of collection (US region) (n = 366). Strain IS-98 STD is used as outgroup to root the tree. US regions are color-coded in the branches of the tree as: Northeast (CT, NJ, NY); green, South (FL, GA, LA, MD, MS, TX); orange; Midwest (IL, MI, NE, ND, SD, WI); blue, and West (AZ, CA, CO, ID, NV, UT); red. Strains collected outside the US (Mexico and Israel) are shown in black. Taxon names at the tip of the branches correspond to GenBank accession codes.
(TIF)

Figure S3 Consensus maximum-likelihood tree of WNV ORF, from the US (1999–2011), by host (n = 366). Strain IS-98 STD is used as outgroup to root the tree. Host-origin is color-coded in the branches of the tree as: mammals other than humans (black), human (red), avian (blue) and mosquito (green). Taxon names at the tip of the branches correspond to GenBank accession codes.
(TIF)

Figure S4 Maximum clade credibility tree derived from the Bayesian analysis of the ORF of WNV strains infecting humans in the US, 1999–2011. WNV genotypes are color-coded in the branches of the tree as NY99 (black), WN02 (blue), SW/WN03 (purple) and cluster MW/WN06 (red). The posterior probability for the nodes in the tree is indicated by a red circle (*P*>0.85).
(TIF)

Figure S5 Consensus maximum-likelihood tree of all available WNV ORF from the US, 1999–2011 (n = 363), in annotated version including spatial and temporal distribution of the different US WNV genotypes, and taxon names at the tip of the branches, corresponding to GenBank accession codes. WNV genotypes are color-coded in the branches of the tree as NY99 (black), intermediate (orange), WN02 (blue), SW/WN03 (purple) and cluster MW/WN06 (red). Nodes containing Hu-WNV sequences within genotype WN02 are shown highlighted in green. Amino acid changes defining important nodes are identified with red arrows. For each genotype, states shown in red in the US map are those from which strains have been sequenced and available for analysis.
(TIF)

Table S1 List of North American WNV strains used in this study, by host, state and year of isolation.
(DOCX)

Table S2 Selection pressure acting upon codons of WNV strains collected in the US (1999–2011), ALL dataset, by host. Open Reading Frame (3,433 codons). Includes codons only detected by MEME.
(DOCX)

Table S3 Selection pressure acting upon codons of WNV strains collected in the US (1999–2011), detected by the methods employed in HyPhy (Datamonkey server). Analysis by individual gene (ALL dataset) in all ORF sequences available (n = 363).
(DOCX)

Table S4 Selection pressure acting upon codons of WNV strains collected in the US (1999–2011), detected by the methods employed in HyPhy (Datamonkey server). Analysis by individual gene in Hu-WNV isolates (n = 61).
(DOCX)

Table S5 Summary of Bayesian estimates of population dynamics of WNV infecting humans in the US (+ strain IS-98 STD, n = 62), calculated using BEAST 1.6.2.
(DOCX)
