# Supplementary figures and images for: Evolutionary Dynamics of West Nile Virus in the United States, 1999–2011: Phylogeny, Selection Pressure and Evolutionary Time-Scale Analysis
Source: PLoS Negl Trop Dis. 2013 May 30;7(5):e2245. doi: 10.1371/journal.pntd.0002245 (PMC3667762; doi:10.1371/journal.pntd.0002245)

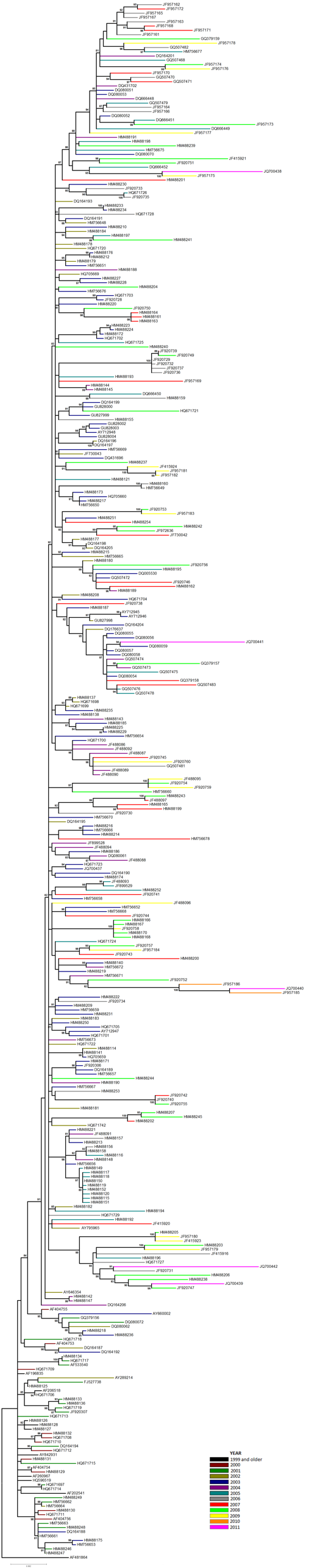

Supplement: Figure S1 — Consensus maximum-likelihood tree of WNV ORF, from the US (1999–2011), by year of collection (n = 366). Strain IS-98 STD is used as outgroup to root the tree. Year of collection is color-coded according to the insert at the bottom of the figure. Taxon names at the tip of the branches correspond to GenBank accession codes. (TIF) [file pntd.0002245.s001.tif]

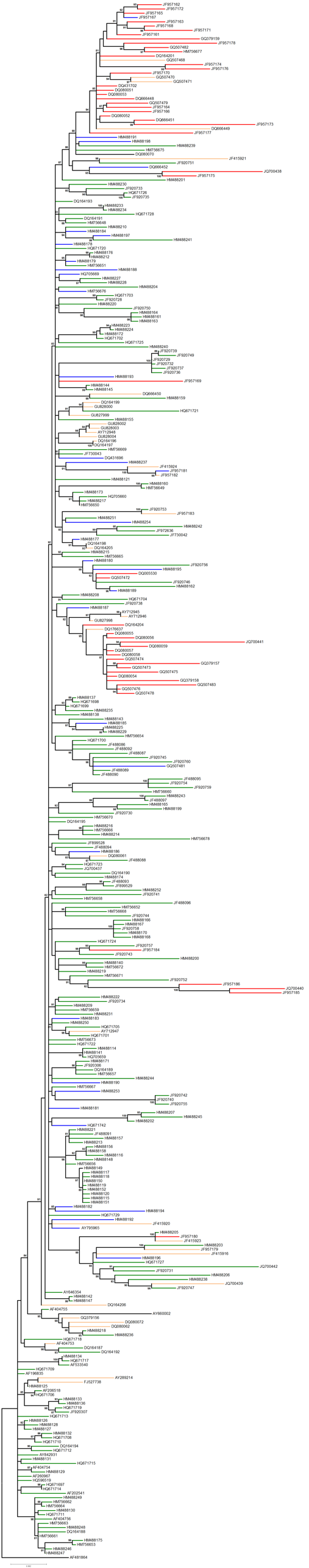

Supplement: Figure S2 — Consensus maximum-likelihood tree of WNV ORF, from the US (1999–2011), by place of collection (US region) (n = 366). Strain IS-98 STD is used as outgroup to root the tree. US regions are color-coded in the branches of the tree as: Northeast (CT, NJ, NY); green, South (FL, GA, LA, MD, MS, TX); orange; Midwest (IL, MI, NE, ND, SD, WI); blue, and West (AZ, CA, CO, ID, NV, UT); red. Strains collected outside the US (Mexico and Israel) are shown in black. Taxon names at the tip of the branches correspond to GenBank accession codes. (TIF) [file pntd.0002245.s002.tif]

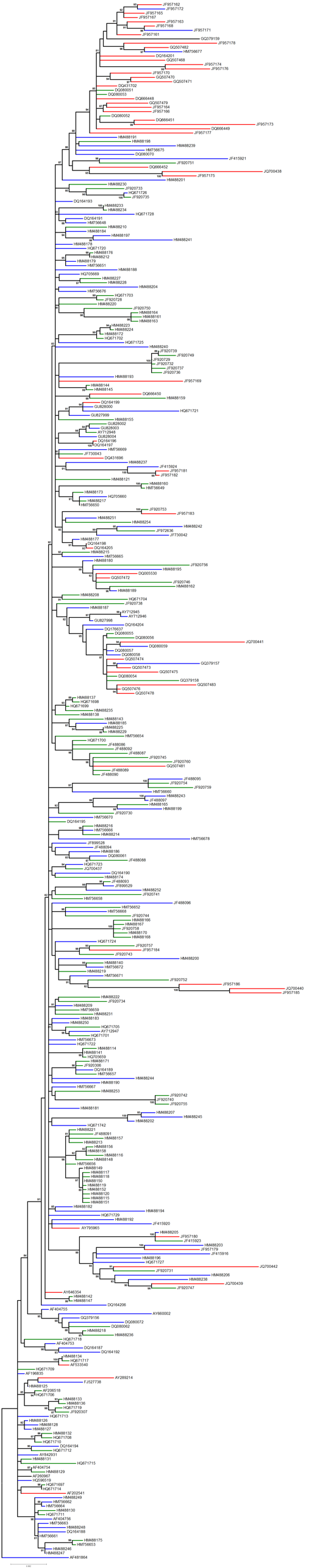

Supplement: Figure S3 — Consensus maximum-likelihood tree of WNV ORF, from the US (1999–2011), by host (n = 366). Strain IS-98 STD is used as outgroup to root the tree. Host-origin is color-coded in the branches of the tree as: mammals other than humans (black), human (red), avian (blue) and mosquito (green). Taxon names at the tip of the branches correspond to GenBank accession codes. (TIF) [file pntd.0002245.s003.tif]

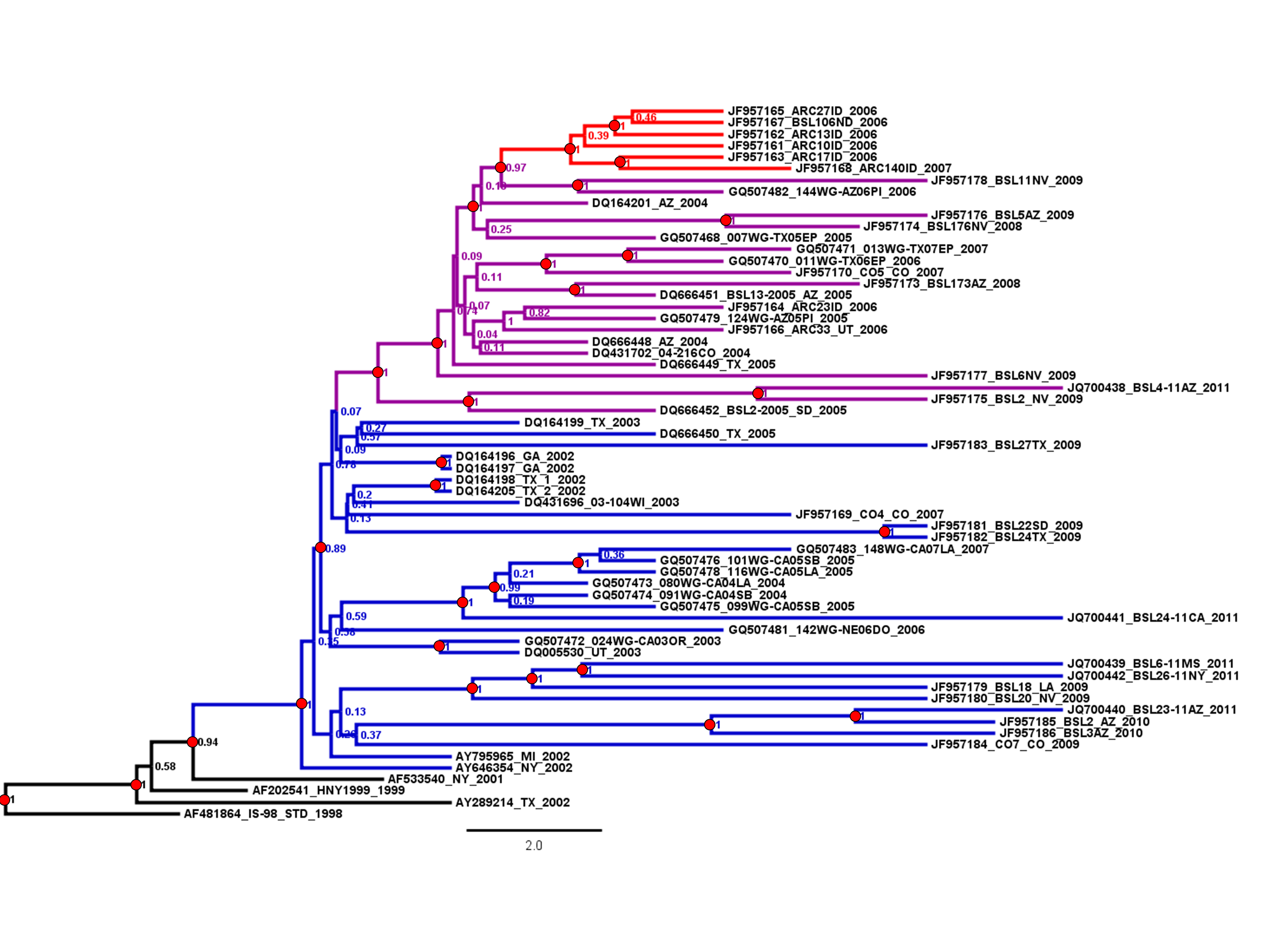

Supplement: Figure S4 — Maximum clade credibility tree derived from the Bayesian analysis of the ORF of WNV strains infecting humans in the US, 1999–2011. WNV genotypes are color-coded in the branches of the tree as NY99 (black), WN02 (blue), SW/WN03 (purple) and cluster MW/WN06 (red). The posterior probability for the nodes in the tree is indicated by a red circle (P>0.85). (TIF) [file pntd.0002245.s004.tif]

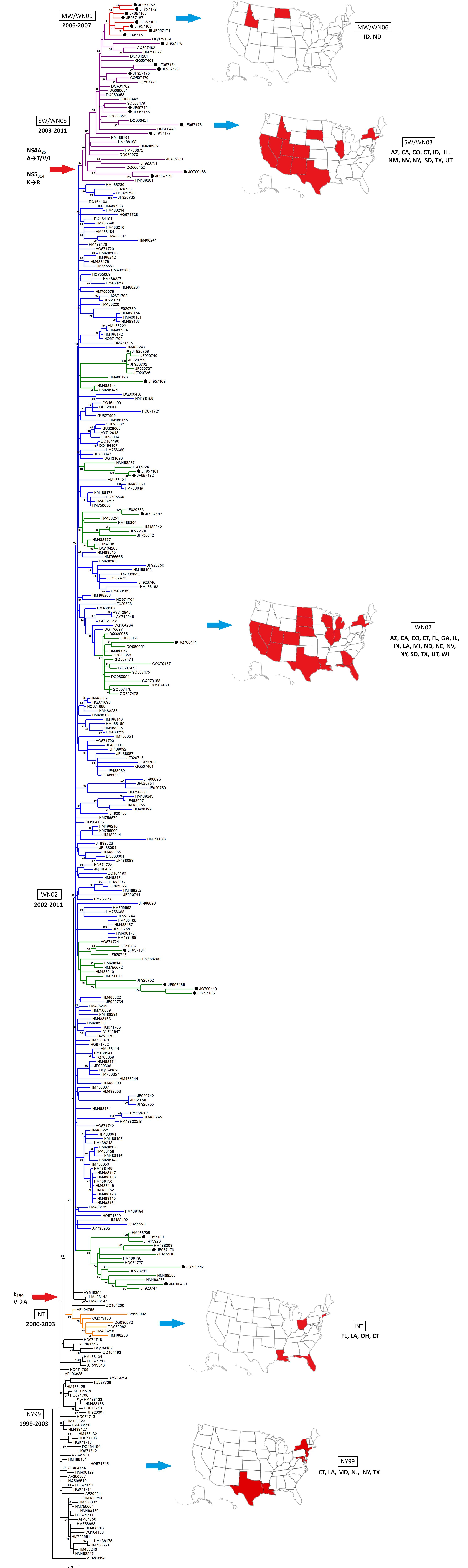

Supplement: Figure S5 — Consensus maximum-likelihood tree of all available WNV ORF from the US, 1999–2011 (n = 363), in annotated version including spatial and temporal distribution of the different US WNV genotypes, and taxon names at the tip of the branches, corresponding to GenBank accession codes. WNV genotypes are color-coded in the branches of the tree as NY99 (black), intermediate (orange), WN02 (blue), SW/WN03 (purple) and cluster MW/WN06 (red). Nodes containing Hu-WNV sequences within genotype WN02 are shown highlighted in green. Amino acid changes defining important nodes are identified with red arrows. For each genotype, states shown in red in the US map are those from which strains have been sequenced and available for analysis. (TIF) [file pntd.0002245.s005.tif]
