# Supplementary material for: Evolutionary Dynamics of West Nile Virus in the United States, 1999–2011: Phylogeny, Selection Pressure and Evolutionary Time-Scale Analysis
Source: PLoS Negl Trop Dis. 2013 May 30;7(5):e2245. doi: 10.1371/journal.pntd.0002245 (PMC3667762; doi:10.1371/journal.pntd.0002245)
Supplement: Table S1 — List of North American WNV strains used in this study, by host, state and year of isolation. (DOCX) [file pntd.0002245.s006.docx]

**Table S1. List of North American WNV strains used in this study, by host, state and year of isolation.**

| # | GenBank accession no. | Strain name | Host | Year of  Isolation | State/  Country |
| --- | --- | --- | --- | --- | --- |
| 1 | AF196835 | NY99-flamingo382-99 | Avian | 1999 | NY |
| 2 | AF202541 | HNY1999 | Human | 1999 | NY |
| 3 | AF206518 | 2741 | Mosquito | 1999 | CT |
| 4 | AF260967 | NY99-eqhs | Horse | 1999 | NY |
| 5 | AY842931 | 385-99 | Avian | 1999 | NY |
| 6 | HM488125 | BID-V4186 | Avian | 1999 | CT |
| 7 | HM488126 | BID-V4187 | Avian | 1999 | CT |
| 8 | HM488127 | BID-V4188 | Avian | 1999 | CT |
| 9 | HM488128 | BID-V4189 | Avian | 1999 | CT |
| 10 | HQ596519 | 4132 | Avian | 1999 | NY |
| 11 | HQ671706 | BID-V4898 | Mosquito | 1999 | CT |
|  |  |  |  |  |  |
| 12 | AF404753 | MD 2000-crow265 | Avian | 2000 | MD |
| 13 | AF404754 | NJ 2000 MQ5488 | Mosquito | 2000 | NJ |
| 14 | AF404755 | NY 2000-grouse3282 | Avian | 2000 | NY |
| 15 | AF404756 | NY 2000-crow3356 | Avian | 2000 | NY |
| 16 | HM488129 | BID-V4191 | Mosquito | 2000 | CT |
| 17 | HM488130 | BID-V4192 | Mosquito | 2000 | CT |
| 18 | HM488131 | BID-V4193 | Mosquito | 2000 | CT |
| 19 | HM488132 | BID-V4194 | Mosquito | 2000 | CT |
| 20 | HQ671708 | BID-V4900 | Mosquito | 2000 | CT |
| 21 | HQ671709 | BID-V4901 | Mosquito | 2000 | CT |
| 22 | HQ671710 | BID-V4902 | Mosquito | 2000 | CT |
| 23 | HQ671711 | BID-V4903 | Mosquito | 2000 | CT |
| 24 | HQ671712 | BID-V4904 | Mosquito | 2000 | CT |
|  |  |  |  |  |  |
| 25 | AF533540 | NY_2001 | Human | 2001 | NY |
| 26 | DQ080072 | FL232 | Avian | 2001 | FL |
| 27 | DQ164194 | NY 2001 Suffolk | Avian | 2001 | NY |
| 28 | FJ527738 | LSU-AR01 | Avian | 2001 | LA |
| 29 | GQ379156 | FL2001_67030 | Avian | 2001 | FL |
| 30 | JF920307 | BID-V4907 | Mosquito | 2001 | CT |
| 31 | HM488133 | BID-V4195 | Mosquito | 2001 | CT |
| 32 | HM488134 | BID-V4198 | Mosquito | 2001 | CT |
| 33 | HM488136 | BID-V4200 | Mosquito | 2001 | CT |
| 34 | HM488246 | BID-V4689 | Avian | 2001 | NY |
| 35 | HM488247 | BID-V4691 | Avian | 2001 | NY |
| 36 | HM488248 | BID-V4694 | Avian | 2001 | NY |
| 37 | HM488249 | BID-V4696 | Avian | 2001 | NY |
| 38 | HM756661 | BID-V4692 | Avian | 2001 | NY |
| 39 | HM756662 | BID-V4693 | Avian | 2001 | NY |
| 40 | HM756663 | BID-V4697 | Avian | 2001 | NY |
| 41 | HQ671697 | BID-V4197 | Mosquito | 2001 | CT |
| 42 | HQ671713 | BID-V4905 | Avian | 2001 | CT |
| 43 | HQ671714 | BID-V4906 | Mosquito | 2001 | CT |
| 44 | HQ671715 | BID-V4908 | Mosquito | 2001 | CT |
| 45 | HQ671717 | BID-V4910 | Mosquito | 2001 | CT |
| 46 | HQ671718 | BID-V4911 | Mosquito | 2001 | CT |
| 47 | HQ671719 | BID-V4912 | Mosquito | 2001 | CT |
|  |  |  |  |  |  |
| 48 | AY289214 | TVP 8533 | Human | 2002 | TX |
| 49 | AY646354 | NY 2002 | Human | 2002 | NY |
| 50 | AY795965 | ARC10 | Human | 2002 | MI |
| 51 | DQ080062 | TWN165 | Mosquito | 2002 | LA |
| 52 | DQ164187 | NY_2002_Broome | Avian | 2002 | NY |
| 53 | DQ164193 | NY_2002_Clinton | Avian | 2002 | NY |
| 54 | DQ164195 | NY_2002_Nassau | Mosquito | 2002 | NY |
| 55 | DQ164196 | GA_2002_1 | Human | 2002 | GA |
| 56 | DQ164197 | GA_2002_2 | Human | 2002 | GA |
| 57 | DQ164198 | TX_2002_1 | Human | 2002 | TX |
| 58 | DQ164205 | TX 2002_2 | Human | 2002 | TX |
| 59 | DQ176637 | TX_2002-HC | Avian | 2002 | TX |
| 60 | GU827998 | Bird114 | Avian | 2002 | TX |
| 61 | JF730043 | BID-V5170 | Mosquito | 2002 | CT |
| 62 | HM488114 | BID-V4102 | Mosquito | 2002 | CT |
| 63 | HM488137 | BID-V4202 | Mosquito | 2002 | CT |
| 64 | HM488177 | BID-V4336 | Avian | 2002 | IL |
| 65 | HM488178 | BID-V4337 | Avian | 2002 | IL |
| 66 | HM488179 | BID-V4338 | Avian | 2002 | IL |
| 67 | HM488180 | BID-V4339 | Avian | 2002 | IL |
| 68 | HM488181 | BID-V4340 | Avian | 2002 | IL |
| 69 | HM488182 | BID-V4341 | Avian | 2002 | IL |
| 70 | HM488183 | BID-V4345 | Avian | 2002 | IL |
| 71 | HM488184 | BID-V4346 | Avian | 2002 | IL |
| 72 | HM488208 | BID-V4204 | Mosquito | 2002 | CT |
| 73 | HM756648 | BID-V4205 | Avian | 2002 | CT |
| 74 | HM756664 | BID-V4701 | Avian | 2002 | NY |
| 75 | HM756665 | BID-V4709 | Avian | 2002 | NY |
| 76 | HQ671698 | BID-V4203 | Mosquito | 2002 | CT |
| 77 | HQ671699 | BID-V4206 | Mosquito | 2002 | CT |
| 78 | HQ671720 | BID-V4913 | Avian | 2002 | CT |
| 79 | HQ671722 | BID-V4704 | Avian | 2002 | NY |
| 80 | HQ671742 | BID-V4343 | Avian | 2002 | IL |
| 81 | HQ705669 | BID-V4342 | Avian | 2002 | IL |
|  |  |  |  |  |  |
| 82 | AY660002 | TM171-03 | Avian | 2003 | Mexico |
| 83 | AY712945 | Bird1153 | Avian | 2003 | TX |
| 84 | AY712946 | Bird1171 | Avian | 2003 | TX |
| 85 | AY712947 | Bird1461 | Avian | 2003 | TX |
| 86 | AY712948 | Mosquito v4369 | Mosquito | 2003 | TX |
| 87 | DQ005530 | BSL5-2003 | Human | 2003 | UT |
| 88 | DQ080051 | A-AZ-03-1623 | Mosquito | 2003 | AZ |
| 89 | DQ080052 | B-AZ-03-1681 | Mosquito | 2003 | AZ |
| 90 | DQ080053 | C-AZ-03_03-1799 | Mosquito | 2003 | AZ |
| 91 | DQ080054 | E-CA-03_GRLA-1260 | Mosquito | 2003 | CA |
| 92 | DQ080055 | F-CA-03_IMPR_102 | Mosquito | 2003 | CA |
| 93 | DQ080056 | G-CA-03_IMPR-1075 | Mosquito | 2003 | CA |
| 94 | DQ080057 | I-CA-03_Arcadia-S0331532 | Avian | 2003 | CA |
| 95 | DQ080058 | J-CA-03_Arcadia-S0334814 | Avian | 2003 | CA |
| 96 | DQ080059 | L-CA-04_SAC-04-7168 | Avian | 2003 | CA |
| 97 | DQ080070 | TVP9115 | Avian | 2003 | Mexico |
| 98 | DQ164190 | NY_2003_Suffolk | Avian | 2003 | NY |
| 99 | DQ164191 | NY_2003_Chautauqua | Avian | 2003 | NY |
| 100 | DQ164192 | NY_2003_Rockland | Avian | 2003 | NY |
| 101 | DQ164188 | NY_2003_Westchester | Avian | 2003 | NY |
| 102 | DQ164189 | NY_2003_Albany | Avian | 2003 | NY |
| 103 | DQ164199 | TX_2003 | Human | 2003 | TX |
| 104 | DQ164204 | CO_2003_1 | Avian | 2003 | CO |
| 105 | DQ431696 | 03-104WI | Human | 2003 | WI |
| 106 | GQ507472 | 024WG-CA03OR | Human | 2003 | CA |
| 107 | GU827999 | Bird1576 | Avian | 2003 | TX |
| 108 | GU828000 | Bird1175 | Avian | 2003 | TX |
| 109 | GU828002 | v4095 | Mosquito | 2003 | TX |
| 110 | GU828003 | Bird1881 | Avian | 2003 | TX |
| 111 | GU828004 | Bird1519 | Avian | 2003 | TX |
| 112 | JF920306 | BID-V4597 | Mosquito | 2003 | CT |
| 113 | JF920728 | BID-V4568 | Mosquito | 2003 | CT |
| 114 | HM488138 | BID-V4207 | Mosquito | 2003 | CT |
| 115 | HM488140 | BID-V4210 | Mosquito | 2003 | CT |
| 116 | HM488141 | BID-V4212 | Mosquito | 2003 | CT |
| 117 | HM488171 | BID-V4560 | Mosquito | 2003 | CT |
| 118 | HM488172 | BID-V4561 | Mosquito | 2003 | CT |
| 119 | HM488173 | BID-V4562 | Mosquito | 2003 | CT |
| 120 | HM488174 | BID-V4563 | Mosquito | 2003 | CT |
| 121 | HM488175 | BID-V4569 | Mosquito | 2003 | CT |
| 122 | HM488176 | BID-V4575 | Mosquito | 2003 | CT |
| 123 | HM488185 | BID-V4347 | Avian | 2003 | IL |
| 124 | HM488186 | BID-V4350 | Avian | 2003 | IL |
| 125 | HM488187 | BID-V4351 | Avian | 2003 | IL |
| 126 | HM488209 | BID-V4564 | Avian | 2003 | CT |
| 127 | HM488210 | BID-V4565 | Mosquito | 2003 | CT |
| 128 | HM488212 | BID-V4567 | Mosquito | 2003 | CT |
| 129 | HM488213 | BID-V4571 | Mosquito | 2003 | CT |
| 130 | HM488214 | BID-V4572 | Mosquito | 2003 | CT |
| 131 | HM488215 | BID-V4573 | Mosquito | 2003 | CT |
| 132 | HM488216 | BID-V4574 | Mosquito | 2003 | CT |
| 133 | HM488217 | BID-V4581 | Mosquito | 2003 | CT |
| 134 | HM488218 | BID-V4583 | Mosquito | 2003 | CT |
| 135 | HM488219 | BID-V4585 | Mosquito | 2003 | CT |
| 136 | HM488220 | BID-V4586 | Mosquito | 2003 | CT |
| 137 | HM488221 | BID-V4593 | Mosquito | 2003 | CT |
| 138 | HM488222 | BID-V4599 | Mosquito | 2003 | CT |
| 139 | HM488223 | BID-V4603 | Mosquito | 2003 | CT |
| 140 | HM488224 | BID-V4604 | Mosquito | 2003 | CT |
| 141 | HM488225 | BID-V4605 | Mosquito | 2003 | CT |
| 142 | HM488227 | BID-V4608 | Mosquito | 2003 | CT |
| 143 | HM488228 | BID-V4609 | Mosquito | 2003 | CT |
| 144 | HM488229 | BID-V4610 | Mosquito | 2003 | CT |
| 145 | HM488230 | BID-V4612 | Mosquito | 2003 | CT |
| 146 | HM488231 | BID-V4613 | Mosquito | 2003 | CT |
| 147 | HM488233 | BID-V4616 | Mosquito | 2003 | CT |
| 148 | HM488234 | BID-V4617 | Mosquito | 2003 | CT |
| 149 | HM488235 | BID-V4619 | Mosquito | 2003 | CT |
| 150 | HM488236 | BID-V4700 | Mosquito | 2003 | CT |
| 151 | HM488250 | BID-V4717 | Avian | 2003 | NY |
| 152 | HM488251 | BID-V4719 | Avian | 2003 | NY |
| 153 | HM756650 | BID-V4582 | Mosquito | 2003 | CT |
| 154 | HM756651 | BID-V4584 | Avian | 2003 | CT |
| 155 | HM756652 | BID-V4587 | Mosquito | 2003 | CT |
| 156 | HM756653 | BID-V4588 | Mosquito | 2003 | CT |
| 157 | HM756654 | BID-V4598 | Mosquito | 2003 | CT |
| 158 | HM756656 | BID-V4615 | Mosquito | 2003 | CT |
| 159 | HM756657 | BID-V4685 | Mosquito | 2003 | CT |
| 160 | HM756658 | BID-V4686 | Mosquito | 2003 | CT |
| 161 | HM756659 | BID-V4687 | Mosquito | 2003 | CT |
| 162 | HM756666 | BID-V4711 | Avian | 2003 | NY |
| 163 | HM756667 | BID-V4712 | Avian | 2003 | NY |
| 164 | HM756668 | BID-V4716 | Avian | 2003 | NY |
| 165 | HM756669 | BID-V4718 | Avian | 2003 | NY |
| 166 | HM756670 | BID-V4720 | Avian | 2003 | NY |
| 167 | HM756676 | BID-V4349 | Avian | 2003 | IL |
| 168 | HQ671700 | BID-V4576 | Mosquito | 2003 | CT |
| 169 | HQ671701 | BID-V4590 | Mosquito | 2003 | CT |
| 170 | HQ671702 | BID-V4595 | Mosquito | 2003 | CT |
| 171 | HQ671703 | BID-V4611 | Mosquito | 2003 | CT |
| 172 | HQ671704 | BID-V4618 | Mosquito | 2003 | CT |
| 173 | HQ671705 | BID-V4620 | Mosquito | 2003 | CT |
| 174 | HQ671723 | BID-V4715 | Avian | 2003 | NY |
| 175 | HQ705659 | BID-V4209 | Mosquito | 2003 | CT |
| 176 | HQ705660 | BID-V4714 | Avian | 2003 | NY |
| 177 | JQ700437* | NY10-03 | Mosquito | 2003 | NY |
|  |  |  |  |  |  |
| 178 | DQ080061 | TWN496 | Avian | 2004 | LA |
| 179 | DQ164201 | AZ_2004 | Human | 2004 | AZ |
| 180 | DQ431702 | 04-216CO | Human | 2004 | CO |
| 181 | DQ666448 | BSL5-2004 | Human | 2004 | AZ |
| 182 | GQ507473 | 080WG-CA04LA | Human | 2004 | CA |
| 183 | GQ507474 | 091WG-CA04SB | Human | 2004 | CA |
| 184 | HM488142 | BID-V4214 | Mosquito | 2004 | CT |
| 185 | HM488143 | BID-V4215 | Mosquito | 2004 | CT |
| 186 | HM488144 | BID-V4216 | Mosquito | 2004 | CT |
| 187 | HM488145 | BID-V4217 | Mosquito | 2004 | CT |
| 188 | HM488147 | BID-V4219 | Mosquito | 2004 | CT |
| 189 | HM488148 | BID-V4220 | Mosquito | 2004 | CT |
| 190 | HM488188 | BID-V4353 | Avian | 2004 | IL |
| 191 | HM488189 | BID-V4367 | Avian | 2004 | IL |
| 192 | HM488190 | BID-V4368 | Avian | 2004 | IL |
| 193 | HM488191 | BID-V4369 | Avian | 2004 | IL |
| 194 | HM756671 | BID-V4798 | Avian | 2004 | NY |
| 195 | HM756672 | BID-V4799 | Avian | 2004 | NY |
| 196 | HM756673 | BID-V4801 | Avian | 2004 | NY |
| 197 | JF488086 | BID-V5176 | Mosquito | 2004 | CT |
| 198 | JF488087 | BID-V5177 | Mosquito | 2004 | CT |
| 199 | JF488088 | BID-V5178 | Mosquito | 2004 | CT |
| 200 | JF488089 | BID-V5179 | Mosquito | 2004 | CT |
| 201 | JF488090 | BID-V5180 | Mosquito | 2004 | CT |
| 202 | JF488091 | BID-V5181 | Mosquito | 2004 | CT |
| 203 | JF488092 | BID-V5182 | Mosquito | 2004 | CT |
| 204 | JF488094 | BID-V5150 | Avian | 2004 | NY |
| 205 | JF899528 | BID-V4800 | Avian | 2004 | NY |
| 206 | DQ164206 | TX_2004 | Avian | 2004 | TX |
|  |  |  |  |  |  |
| 207 | DQ666449 | GCTX1-2005 | Human | 2005 | TX |
| 208 | DQ666450 | GCTX2-2005 | Human | 2005 | TX |
| 209 | DQ666451 | BSL13-2005 | Human | 2005 | AZ |
| 210 | DQ666452 | BSL2-2005 | Human | 2005 | SD |
| 211 | GQ507468 | 007WG-TX05EP | Human | 2005 | TX |
| 212 | GQ507475 | 099WG-CA05SB | Human | 2005 | CA |
| 213 | GQ507476 | 101WG-CA05SB | Human | 2005 | CA |
| 214 | GQ507478 | 116WG-CA05LA | Human | 2005 | CA |
| 215 | GQ507479 | 124WG-AZ05PI | Human | 2005 | AZ |
| 216 | HM488115 | BID-V4103 | Mosquito | 2005 | CT |
| 217 | HM488116 | BID-V4104 | Mosquito | 2005 | CT |
| 218 | HM488117 | BID-V4105 | Mosquito | 2005 | CT |
| 219 | HM488118 | BID-V4107 | Mosquito | 2005 | CT |
| 220 | HM488119 | BID-V4108 | Mosquito | 2005 | CT |
| 221 | HM488120 | BID-V4109 | Mosquito | 2005 | CT |
| 222 | HM488121 | BID-V4110 | Mosquito | 2005 | CT |
| 223 | HM488149 | BID-V4223 | Mosquito | 2005 | CT |
| 224 | HM488150 | BID-V4224 | Mosquito | 2005 | CT |
| 225 | HM488151 | BID-V4225 | Mosquito | 2005 | CT |
| 226 | HM488152 | BID-V4226 | Mosquito | 2005 | CT |
| 227 | HM488192 | BID-V4371 | Avian | 2005 | IL |
| 228 | HM488193 | BID-V4373 | Avian | 2005 | IL |
| 229 | HM488194 | BID-V4374 | Avian | 2005 | IL |
| 230 | HM488195 | BID-V4375 | Avian | 2005 | IL |
| 231 | HM488196 | BID-V4376 | Avian | 2005 | IL |
| 232 | HM488197 | BID-V4377 | Avian | 2005 | IL |
| 233 | HM488198 | BID-V4378 | Mosquito | 2005 | IL |
| 234 | HM488252 | BID-V4805 | Avian | 2005 | NY |
| 235 | HM756675 | BID-V4806 | Avian | 2005 | NY |
| 236 | HM756677 | BID-V4530 | Avian | 2005 | NM |
| 237 | HQ671724 | BID-V4883 | Avian | 2005 | NY |
| 238 | HQ671725 | BID-V4885 | Avian | 2005 | NY |
| 239 | HQ671726 | BID-V4887 | Avian | 2005 | NY |
| 240 | JF488093 | BID-V5188 | Mosquito | 2005 | CT |
| 241 | JF899529 | BID-V4808 | Avian | 2005 | NY |
|  |  |  |  |  |  |
| 242 | GQ507470 | 011WG-TX06EP | Human | 2006 | TX |
| 243 | GQ507481 | 142WG-NE06DO | Human | 2006 | NE |
| 244 | GQ507482 | 144WG-AZ06PI | Human | 2006 | AZ |
| 245 | HM488155 | BID-V4229 | Mosquito | 2006 | CT |
| 246 | HM488156 | BID-V4230 | Mosquito | 2006 | CT |
| 247 | HM488157 | BID-V4231 | Mosquito | 2006 | CT |
| 248 | HM488158 | BID-V4232 | Mosquito | 2006 | CT |
| 249 | HM488159 | BID-V4233 | Mosquito | 2006 | CT |
| 250 | HM488160 | BID-V4355 | Mosquito | 2006 | CT |
| 251 | HM488253 | BID-V4553 | Mosquito | 2006 | IL |
| 252 | HM756649 | BID-V4354 | Mosquito | 2006 | CT |
| 253 | HQ671727 | BID-V4889 | Avian | 2006 | NY |
| 254 | HQ671728 | BID-V4891 | Avian | 2006 | NY |
| 255 | HQ671729 | BID-V4892 | Avian | 2006 | NY |
| 256 | JF415916 | TX6276 | Avian | 2006 | TX |
| 257 | JF920729 | BID-V5196 | Mosquito | 2006 | CT |
| 258 | JF920730 | BID-V5197 | Mosquito | 2006 | CT |
| 259 | JF920731 | BID-V5201 | Mosquito | 2006 | CT |
| 260 | JF920732 | BID-V5202 | Mosquito | 2006 | CT |
| 261 | JF920733 | BID-V5203 | Mosquito | 2006 | CT |
| 262 | JF920734 | BID-V5204 | Mosquito | 2006 | CT |
| 263 | JF920735 | BID-V5205 | Mosquito | 2006 | CT |
| 264 | JF920736 | BID-V5206 | Mosquito | 2006 | CT |
| 265 | JF920737 | BID-V5207 | Mosquito | 2006 | CT |
| 266 | JF957161* | ARC10-06 | Human | 2006 | ID |
| 267 | JF957162* | ARC13-06 | Human | 2006 | ID |
| 268 | JF957163* | ARC17-06 | Human | 2006 | ID |
| 269 | JF957164* | ARC23-06 | Human | 2006 | ID |
| 270 | JF957165* | ARC27-06 | Human | 2006 | ID |
| 271 | JF957166* | ARC33-06 | Human | 2006 | UT |
| 272 | JF957167* | BSL106-06 | Human | 2006 | ND |
|  |  |  |  |  |  |
| 273 | GQ379158 | ORCO0559-07 | Mosquito | 2007 | CA |
| 274 | GQ507471 | 013WG-TX07EP | Human | 2007 | TX |
| 275 | GQ507483 | 148WG-CA07LA | Human | 2007 | CA |
| 276 | HM488161 | BID-V4356 | Mosquito | 2007 | CT |
| 277 | HM488162 | BID-V4357 | Mosquito | 2007 | CT |
| 278 | HM488163 | BID-V4359 | Mosquito | 2007 | CT |
| 279 | HM488164 | BID-V4360 | Mosquito | 2007 | CT |
| 280 | HM488165 | BID-V4361 | Mosquito | 2007 | CT |
| 281 | HM488199 | BID-V4090 | Avian | 2007 | NY |
| 282 | HM488200 | BID-V4092 | Avian | 2007 | NY |
| 283 | HM488201 | BID-V4093 | Avian | 2007 | NY |
| 284 | HM488202 | BID-V4094 | Avian | 2007 | NY |
| 285 | HM488254 | BID-V4559 | Mosquito | 2007 | IL |
| 286 | HM756678 | BID-V4095 | Avian | 2007 | NY |
| 287 | JF415920 | TX7191 | Avian | 2007 | TX |
| 288 | JF488097 | BID-V5148 | Avian | 2007 | NY |
| 289 | JF730042 | BID-V5147 | Avian | 2007 | NY |
| 290 | JF920738 | BID-V5208 | Mosquito | 2007 | CT |
| 291 | JF920739 | BID-V5209 | Mosquito | 2007 | CT |
| 292 | JF920740 | BID-V5210 | Mosquito | 2007 | CT |
| 293 | JF920741 | BID-V5212 | Mosquito | 2007 | CT |
| 294 | JF920742 | BID-V5213 | Mosquito | 2007 | CT |
| 295 | JF920743 | BID-V5214 | Mosquito | 2007 | CT |
| 296 | JF920744 | BID-V5215 | Mosquito | 2007 | CT |
| 297 | JF920745 | BID-V5216 | Mosquito | 2007 | CT |
| 298 | JF920746 | BID-V5217 | Mosquito | 2007 | CT |
| 299 | JF957168* | ARC140-07 | Human | 2007 | ID |
| 300 | JF957169* | CO4-07 | Human | 2007 | CO |
| 301 | JF957170* | CO5-07 | Human | 2007 | CO |
| 302 | JF957171* | ID21_bird | Avian | 2007 | ID |
| 303 | JF957172* | ID28_bird | Avian | 2007 | ID |
|  |  |  |  |  |  |
| 304 | GQ379157 | DB080718-14 | Avian | 2008 | CA |
| 305 | GQ379159 | JPW080813-01 | Squirrel | 2008 | CA |
| 306 | HM488166 | BID-V4362 | Mosquito | 2008 | CT |
| 307 | HM488167 | BID-V4363 | Mosquito | 2008 | CT |
| 308 | HM488168 | BID-V4364 | Mosquito | 2008 | CT |
| 309 | HM488170 | BID-V4366 | Mosquito | 2008 | CT |
| 310 | HM488203 | BID-V4096 | Avian | 2008 | NY |
| 311 | HM488204 | BID-V4098 | Avian | 2008 | NY |
| 312 | HM488205 | BID-V4099 | Avian | 2008 | NY |
| 313 | HM488206 | BID-V4100 | Avian | 2008 | NY |
| 314 | HM488207 | BID-V4101 | Avian | 2008 | NY |
| 315 | HM488237 | BID-V4622 | Avian | 2008 | NY |
| 316 | HM488238 | BID-V4623 | Avian | 2008 | NY |
| 317 | HM488239 | BID-V4624 | Avian | 2008 | NY |
| 318 | HM488240 | BID-V4627 | Avian | 2008 | NY |
| 319 | HM488241 | BID-V4628 | Avian | 2008 | NY |
| 320 | HM488242 | BID-V4631 | Avian | 2008 | NY |
| 321 | HM488243 | BID-V4632 | Avian | 2008 | NY |
| 322 | HM488244 | BID-V4634 | Avian | 2008 | NY |
| 323 | HM488245 | BID-V4635 | Avian | 2008 | NY |
| 324 | HM756660 | BID-V4097 | Avian | 2008 | NY |
| 325 | HQ671721 | BID-V4625 | Avian | 2008 | NY |
| 326 | JF415921 | TX7558 | Avian | 2008 | TX |
| 327 | JF920747 | BID-V5218 | Mosquito | 2008 | CT |
| 328 | JF920749 | BID-V5220 | Mosquito | 2008 | CT |
| 329 | JF920750 | BID-V5222 | Mosquito | 2008 | CT |
| 330 | JF920751 | BID-V5223 | Mosquito | 2008 | CT |
| 331 | JF920752 | BID-V5224 | Mosquito | 2008 | CT |
| 332 | JF920753 | BID-V5225 | Mosquito | 2008 | CT |
| 333 | JF920754 | BID-V5226 | Mosquito | 2008 | CT |
| 334 | JF920755 | BID-V5227 | Mosquito | 2008 | CT |
| 335 | JF920756 | BID-V5229 | Mosquito | 2008 | CT |
| 336 | JF920757 | BID-V5230 | Mosquito | 2008 | CT |
| 337 | JF972636 | BID-V5228 | Mosquito | 2008 | CT |
| 338 | JF957173* | BSL173-08 | Human | 2008 | AZ |
| 339 | JF957174* | BSL176-08 | Human | 2008 | NV |
|  |  |  |  |  |  |
| 340 | JF415923 | M37906 | Mosquito | 2009 | TX |
| 341 | JF415924 | TX7827 | Avian | 2009 | TX |
| 342 | JF488095 | BID-V5157 | Avian | 2009 | NY |
| 343 | JF488096 | BID-V5159 | Avian | 2009 | NY |
| 344 | JF920758 | BID-V5233 | Mosquito | 2009 | CT |
| 345 | JF920759 | BID-V5234 | Mosquito | 2009 | CT |
| 346 | JF920760 | BID-V5235 | Mosquito | 2009 | CT |
| 347 | JF957175* | BSL2-09 | Human | 2009 | NV |
| 348 | JF957176* | BSL5-09 | Human | 2009 | AZ |
| 349 | JF957177* | BSL6-09 | Human | 2009 | NV |
| 350 | JF957178* | BSL11-09 | Human | 2009 | NV |
| 351 | JF957179* | BSL18-09 | Human | 2009 | LA |
| 352 | JF957180* | BSL20-09 | Human | 2009 | NV |
| 353 | JF957181* | BSL22-09 | Human | 2009 | SD |
| 354 | JF957182* | BSL24-09 | Human | 2009 | TX |
| 355 | JF957183* | BSL27-09 | Human | 2009 | TX |
| 356 | JF957184* | CO7-09 | Human | 2009 | CO |
| 357 |  |  |  |  |  |
| 358 | JF957185* | BSL2-10 | Human | 2010 | AZ |
| 359 | JF957186* | BSL3-10 | Human | 2010 | AZ |
| 360 |  |  |  |  |  |
| 361 | JQ700438* | BSL4-11 | Human | 2011 | AZ |
| 362 | JQ700439* | BSL6-11 | Human | 2011 | MS |
| 363 | JQ700440* | BSL23-11 | Human | 2011 | AZ |
| 364 | JQ700441* | BSL24-11 | Human | 2011 | CA |
| 365 | JQ700442* | BSL26-11 | Human | 2011 | NY |
|  |  |  |  |  |  |
| 366 | AF481864 | IS-98 STD | Avian | 1998 | Israel |

* Strain sequenced in this manuscript
