# Supplementary material for: Evolutionary Dynamics of West Nile Virus in the United States, 1999–2011: Phylogeny, Selection Pressure and Evolutionary Time-Scale Analysis
Source: PLoS Negl Trop Dis. 2013 May 30;7(5):e2245. doi: 10.1371/journal.pntd.0002245 (PMC3667762; doi:10.1371/journal.pntd.0002245)
Supplement: Table S2 — Selection pressure acting upon codons of WNV strains collected in the US (1999–2011), ALL dataset, by host. Open Reading Frame (3,433 codons). Includes codons only detected by MEME. (DOCX) [file pntd.0002245.s007.docx]

**Table S2.** **Selection pressure acting upon codons of WNV strains collected in the US (1999-2011), by host.**

| **Dataset** | **Codon** | **Protein**  **and AA #** | **Methods** | | | |  |
| --- | --- | --- | --- | --- | --- | --- | --- |
|  |  |  | **FEL** | **IFEL** | **SLAC** | **MEME** | **MEME only** |
|  |  |  | ***P* value** | | | |  |
| A (n=133), ω: 0.079  462 negatively selected sites |  |  |  |  |  |  |  |
| 1 | 1262 | NS2A_119_ | 0.02 | 0.72 | 0.21 | 0.02 |  |
| 2 | 1493 | NS2B_119_ | 0.33 | *0.14* | 0.45 | 0.04 | **√** |
| 3 | **2209*** | **NS4A-A_85_T** | **0.02** | **0.05** | **0.13** | **0.02** |  |
| 4 | 2320 | NS4B_47_ | (0.67) | (0.47) | 0.67 | **0.02** | **√** |
| 5 | 2363 | NS4B_90_ | 0.06 | 1 | 0.48 | 0.06 |  |
| 6 | 2517 | NS4B_244_ | (0.09) | (0.22) | (0.99) | 0.04 | **√** |
| 7 | 2733 | NS5_205_ | (0.70) | (0.50) | (0.89) | 0.06 | **√** |
| 8 | 2734 | NS5_206_ | (0.58) | (0.36) | 0.57 | 0.01 | **√** |
| 9 | 3216 | NS5_688_ | (0.83) | (0.56) | (0.89) | 0.02 | **√** |
|  |  |  |  |  |  |  |  |
| M (n=167), ω: 0.102  361 negatively selected sites |  |  |  |  |  |  |  |
| 1 | 489 | E_199_ | 0.07 | 1 | 0.48 | 0.07 |  |
| 2 | 1031 | NS1_240_ | 0.09 | 1 | 0.47 | 0.09 |  |
| 3 | 1474 | NS2B_100_ | (0.63) | (0.34) | (0.89) | 0.02 | **√** |
| 4 | 1860 | NS3_355_ | 0.07 | 1 | 0.31 | 0.07 |  |
| 5 | 1941 | NS3_436_ | (0.33) | (0.21) | (0.89) | 0.08 | **√** |
| 6 | **2209*** | **NS4A-A_85_T** | **0.04** | **0.02** | *0.19* | **0.04** |  |
| 7 | **2259** | **NS4A_135_** | **0.07** | *0.13* | **0.04** | **0.06** |  |
| 8 | 2393 | NS4B_120_ | (0.79) | (0.40) | (0.90) | 0.008 | **√** |
| 9 | 2842 | NS5_314_ | *0.12* | 0.08 | 0.24 | 0.01 | **√** |
| 10 | 3234 | NS5_706_ | 0.10 | 1 | 0.47 | 0.10 |  |
|  |  |  |  |  |  |  |  |
| H (n=61), ω: 0.127  231 negatively selected sites |  |  |  |  |  |  |  |
| 1 | 999 | NS1_208_ | 0.07 | 1 | 0.49 | 0.07 |  |
| 2 | 1438 | NS2B_64_ | 0.08 | 1 | 0.48 | 0.08 |  |
| 3 | **1841** | **NS3_336_** | **0.10** | **0.07** | *0.17* | **0.10** |  |
| 4 | **2209*** | **NS4A-A_85_T** | **0.07** | **0.02** | **0.30** | **0.07** |  |
| 5 | 2842 | NS5_314_ | *0.12* | 0.04 | 0.22 | 0.06 |  |
| 6 | 3132 | NS5_604_ | 0.09 | 1 | 0.49 | 0.09 |  |
| 7 | 3240 | NS5_712_ | 0.26 | 1 | 0.30 | 0.004 | **√** |
|  |  |  |  |  |  |  |  |
| A+M (n=300), ω: 0.091  769 negatively selected sites |  |  |  |  |  |  |  |
| 1 | 468 | E_178_ | (0.55) | 0.54 | (0.73) | 0.02 | **√** |
| 2 | 800 | NS1_9_ | 0.09 | 1 | 0.46 | 0.09 |  |
| 3 | 938 | NS1_147_ | 0.07 | 1 | 0.48 | 0.07 |  |
| 4 | 957 | NS1_166_ | (0.45) | (0.31) | (0.88) | 0.09 | **√** |
| 5 | 1031 | NS1_240_ | 0.08 | 1 | 0.46 | 0.08 |  |
| 6 | 1087 | NS1_296_ | (0.72) | (0.42) | (0.89) | 0.01 | **√** |
| 7 | **1262** | **NS2A_119_** | **0.01** | **1** | **0.10** | **0.01** |  |
| 8 | 1474 | NS2B_100_ | (0.24) | (0.22) | (0.96) | 0.02 | **√** |
| 9 | 1941 | NS3_436_ | (0.32) | (0.24) | (0.89) | 0.07 | **√** |
| 10 | **2209*** | **NS4A-A_85_T** | **0.005** | **0.01** | **0.06** | **0.005** |  |
| 11 | 2259 | NS4A_135_ | 0.60 | 0.35 | *0.15* | 0.04 | **√** |
| 12 | 2320 | NS4B_47_ | (0.03) | (0.04) | (0.93) | 0.09 | **√** |
| 13 | 2393 | NS4B_120_ | (0.72) | (0.29) | (0.90) | 0.01 | **√** |
| 14 | 2517 | NS4B_244_ | (0.09) | (0.80) | (0.99) | 0.02 | **√** |
| 15 | 2733 | NS5_205_ | (0.28) | (0.24) | (0.96) | 0.10 | **√** |
| 16 | 2734 | NS5_206_ | (0.58) | (0.25) | 0.57 | 0.006 | **√** |
| 17 | 2842 | NS5_314_ | *0.12* | 0.06 | 0.24 | 0.01 | **√** |
| 18 | 3216 | NS5_688_ | (0.39) | (0.30) | (0.96) | 0.03 | **√** |
|  |  |  |  |  |  |  |  |
| ALL (n=363), ω: 0.105  969 negatively selected sites |  |  |  |  |  |  |  |
| 1 | 447 | E_157_ | (0.33) | (0.24) | (0.89) | 0.02 | **√** |
| 2 | 468 | E_178_ | (0.16) | 0.96 | (0.89) | 0.02 | **√** |
| 3 | 800 | NS1_9_ | 0.04 | 1 | 0.32 | 0.04 |  |
| 4 | **938** | **NS1_147_** | **0.07** | **0.05** | **0.49** | **0.07** |  |
| 5 | 957 | NS1_166_ | (0.46) | (0.31) | (0.88) | 0.09 | **√** |
| 6 | 1031 | NS1_240_ | 0.04 | 1 | 0.32 | 0.03 |  |
| 7 | 1087 | NS1_296_ | (0.72) | (0.42) | (0.89) | 0.01 | **√** |
| 8 | 1108 | NS1_317_ | (0.25) | (0.22) | (0.96) | 0.06 | **√** |
| 9 | 1262 | NS2A_119_ | 0.008 | 1 | 0.10 | 0.008 |  |
| 10 | 1438 | NS2B_64_ | 0.008 | 1 | 0.49 | 0.08 |  |
| 11 | 1474 | NS2B_100_ | (0.09) | (0.13) | (0.99) | 0.03 | **√** |
| 12 | **1841** | **NS3_336_** | **0.09** | **0.07** | *0.16* | **0.09** |  |
| 13 | 1941 | NS3_436_ | (0.33) | (0.24) | (0.89) | 0.07 | **√** |
| 14 | 1959 | NS3_454_ | (0.23) | (0.23) | (0.99) | 0.09 | **√** |
| 15 | **2209*** | **NS4A-A_85_T** | **0.002** | **0.01** | **0.03** | **0.002** |  |
| 16 | 2259 | NS4A_135_ | 0.56 | 0.34 | *0.15* | 0.04 | **√** |
| 17 | 2320 | NS4B_47_ | (0.03) | (0.04) | (0.93) | 0.06 | **√** |
| 18 | 2393 | NS4B_120_ | (0.70) | (0.28) | (0.90) | 0.008 | **√** |
| 19 | 2517 | NS4B_244_ | (0.09) | (0.77) | (0.99) | 0.02 | **√** |
| 20 | 2734 | NS5_206_ | (0.58) | (0.24) | 0.57 | 0.005 | **√** |
| 21 | **2842** | **NS5_314_** | **0.04** | **0.02** | **0.08** | **0.01** |  |
| 22 | 3132 | NS5_604_ | 0.08 | 1 | 0.49 | 0.08 |  |
| 23 | 3216 | NS5_688_ | (0.18) | (0.19) | (0.99) | 0.03 | **√** |

Table represent the analysis of the datasets from each host (A=avian, M=mosquito, A+M=avian + mosquito, H=human, ALL=all hosts origin). Open Reading Frame (3,433 codons). Includes codons only detected by MEME.
* Codon recognized in all datasets
in bold, codons recognized by three or four methods, with statistical significance

ω = dN/dS ratio
FEL = Fixed effects likelihood, IFEL = Internal Fixed effects likelihood, SLAC = Single-likelihood ancestor counting, MEME = Mixed Effects Model of Evolution.

Numbers between parentheses represent codons detected as negatively selected (with or without significance). Numbers in italics represent codons detected positively detected, not significantly, but close to *p* threshold (0.1)
