# Supplementary material for: Evolutionary Dynamics of West Nile Virus in the United States, 1999–2011: Phylogeny, Selection Pressure and Evolutionary Time-Scale Analysis
Source: PLoS Negl Trop Dis. 2013 May 30;7(5):e2245. doi: 10.1371/journal.pntd.0002245 (PMC3667762; doi:10.1371/journal.pntd.0002245)
Supplement: Table S3 — Selection pressure acting upon codons of WNV strains collected in the US (1999–2011), detected by the methods employed in HyPhy (Datamonkey server). Analysis by individual gene (ALL dataset) in all ORF sequences available (n = 363). (DOCX) [file pntd.0002245.s008.docx]

**Table S3.** **Selection pressure acting upon codons (individual genes) of WNV strains collected in the US (1999-2011).**

| **Gene^a^** | **Codon** | **Methods^c^** | | | |
| --- | --- | --- | --- | --- | --- |
|  |  | **FEL** | **IFEL** | **SLAC** | **MEME** |
|  |  | ***P* value** | | | |
|  |  |  |  |  |  |
| NS1 (n=224), codons: 352 , ω^b^: 0.153  75 negatively selected sites |  |  |  |  |  |
| 1 | S_9_N/T | 0.04 | 1 | 0.34 | 0.04 |
| 2 | **L_111_F** | **0.09** | **0.07** | *0.16* | **0.09** |
| 3 | **N_147_D/S** | **0.07** | **0.05** | 0.48 | **0.07** |
| 4 | D_240_N/E | 0.04 | 1 | 0.34 | 0.04 |
| 5 | **Q_324_R/K** | **0.08** | **0.10** | 0.20 | **0.08** |
|  |  |  |  |  |  |
|  |  |  |  |  |  |
|  |  |  |  |  |  |
| NS2A (n=201), codons: 231 , ω^b^: 0.095  67 negatively selected sites |  |  |  |  |  |
| 1 | H_119_Y | 0.04 | *0.11* | 0.23 | 0.04 |
| 2 | R_188_K | 0.08 | *0.19* | *0.18* | 0.08 |
|  |  |  |  |  |  |
|  |  |  |  |  |  |
| NS3 (n=294), codons: 619, ω^b^: 0.077  185 negatively selected sites |  |  |  |  |  |
| 1 | L_336_S | 0.09 | 1 | *0.14* | 0.09 |
|  |  |  |  |  |  |
|  |  |  |  |  |  |
| NS4A (n=134), codons: 149 , ω^b^: 0.109  33 negatively selected sites |  |  |  |  |  |
| 1 | **A_85_T/V/I** | **0.04** | **0.01** | **0.06** | **0.04** |
|  |  |  |  |  |  |
| NS4B (n=223), codons: 255, ω^b^: 0.127  80 negatively selected sites |  |  |  |  |  |
| 1 | G_104_E | 0.10 | 1 | 0.29 | 0.10 |
|  |  |  |  |  |  |
| NS5 (n=320), codons: 905, ω: 0.104  231 negatively selected sites |  |  |  |  |  |
| 1 | **K_314_R** | **0.05** | **0.01** | **0.09** | **0.05** |
| 2 | S_604_R | 0.06 | 1 | 0.48 | 0.06 |
| 3 | Y_706_C/H | 0.09 | 1 | 0.47 | 0.09 |

Table represent the analysis of the individual genes of WNV for the ALL dataset (n=363), detected by the methods employed in HyPhy (Datamonkey server).
^a^ number of sequences differ from the input (n=363), after removal of identical sequences by the server
^b^ ω = dN/dS ratio

^c^ FEL = Fixed effects likelihood, IFEL = Internal Fixed effects likelihood, SLAC = Single-likelihood ancestor counting, MEME = Mixed Effects Model of Evolution. All codons present in the table are recognized by at least two methods. In bold, codons recognized by three or four methods, with statistical significance. *p* values in italics represent codons detected to be under positive selection, not significantly, but close to *p* threshold (0.1).
