# Supplementary material for: Evolutionary Dynamics of West Nile Virus in the United States, 1999–2011: Phylogeny, Selection Pressure and Evolutionary Time-Scale Analysis
Source: PLoS Negl Trop Dis. 2013 May 30;7(5):e2245. doi: 10.1371/journal.pntd.0002245 (PMC3667762; doi:10.1371/journal.pntd.0002245)
Supplement: Table S4 — Selection pressure acting upon codons of WNV strains collected in the US (1999–2011), detected by the methods employed in HyPhy (Datamonkey server). Analysis by individual gene in Hu-WNV isolates (n = 61). (DOCX) [file pntd.0002245.s009.docx]

**Table S4.** **Selection pressure acting upon codons (individual genes) of Hu-WNV strains collected in the US (1999-2011).**

| **Gene^a^** | **Codon** | **Methods^c^** | | | | |
| --- | --- | --- | --- | --- | --- | --- |
|  |  | **FEL** | **IFEL** | **SLAC** | **MEME** | **REL** |
|  |  | ***P* value** | | | | **BF** |
| E (n=54), codons: 501 , ω^b^: 0.110  39 negatively selected sites |  |  |  |  |  |  |
| 1 | N_8_S | 0.07 | 1 | 0.47 | 0.07 | 1 |
|  |  |  |  |  |  |  |
| NS1 (n=49), codons: 352 , ω^b^: 0.156  17 negatively selected sites |  |  |  |  |  |  |
| 1 | D_208_E/N | 0.08 | 1 | 0.49 | 0.08 | 1 |
|  |  |  |  |  |  |  |
| NS3 (n=54), codons: 619, ω^b^: 0.095  39 negatively selected sites |  |  |  |  |  |  |
| 1 | L_336_S | 0.09 | 1 | *0.15* | 0.09 | 1 |
|  |  |  |  |  |  |  |
|  |  |  |  |  |  |  |
| NS4A (n=39), codons: 149 , ω^b^: 0.097  8 negatively selected sites |  |  |  |  |  |  |
| 1 | A_85_T/I^d^ | *0.12* | 0.03 | 0.29 | *0.12* | 3781 |
|  |  |  |  |  |  |  |
|  |  |  |  |  |  |  |
| NS5 (n=58), codons: 905, ω^b^: 0.137  62 negatively selected sites |  |  |  |  |  |  |
| 1 | K_194_R | 0.30 | *0.19* | 0.52 | 0.30 | 439 |
| 2 | K_314_R | *0.14* | 0.02 | 0.26 | *0.13* | 49528 |
| 3 | **S_604_R** | **0.06** | **1** | 0.49 | **0.06** | **388** |
| 4 | P_712_Q | 0.33 | 1 | 0.30 | 0.004 | 2540 |
| 5 | **A_860_T** | *0.16* | **0.10** | 0.44 | **0.06** | **156** |

Table represent the analysis of the individual genes of WNV for the H dataset (n=61), detected by the methods employed in HyPhy (Datamonkey server).
^a^ number of sequences differ from the input (n=61), after removal of identical sequences by the server.

^b^ ω = dN/dS ratio

^c^ FEL = Fixed effects likelihood, IFEL = Internal Fixed effects likelihood, SLAC = Single-likelihood ancestor counting, MEME = Mixed Effects Model of Evolution, REL = Random Effects Likelihood, BF = Bayes Factor. All codons present in the table are recognized by at least two methods. In bold, codons recognized by three or four methods, with statistical significance. *p* values in italics represent codons detected to be under positive selection, not significantly, but close to *p* threshold (0.1).

^d^ Site NS4A_85_ detected also under positive selection pressure by the Bayesian empirical method employed by Selecton (Stern A, Doron-Faigenboim A, Erez E, Martz E, Bacharach E, et al. (2007). Selecton 2007: advanced models for detecting positive and purifying selection using a Bayesian inference approach. Nucleic Acids Res 35(Web Server issue):W506–11).
