# Supplementary material for: Evolutionary Dynamics of West Nile Virus in the United States, 1999–2011: Phylogeny, Selection Pressure and Evolutionary Time-Scale Analysis
Source: PLoS Negl Trop Dis. 2013 May 30;7(5):e2245. doi: 10.1371/journal.pntd.0002245 (PMC3667762; doi:10.1371/journal.pntd.0002245)
Supplement: Table S5 — Summary of Bayesian estimates of population dynamics of WNV infecting humans in the US (+ strain IS-98 STD, n = 62), calculated using BEAST 1.6.2. (DOCX) [file pntd.0002245.s010.docx]

**Table S5.** **Summary of Bayesian estimates of population dynamics of WNV in humans in the US.**

| Molecular clock and  demographic model | Marginal Likelihood, mean | Mean substitution rate, (10^-4^ substitutions/site/year) | Mean age of *tMRCA*, years |
| --- | --- | --- | --- |
| S |  |  |  |
| BSP | -23507.39 | 4.84 | 15.82 |
| Constant | -23511.75 | 4.80 | 16.25 |
| Expansion | -23509.72 | 4.74 | 16.11 |
| Exponential | -23509.69 | 4.73 | 15.99 |
| Logistic | -20509.47 | 4.72 | 16.06 |
|  |  |  |  |
| UCLN |  |  |  |
| **BSP** | **-23482.95** | **5.06** | **15.57** |
| Constant | -23485.52 | 5.09 | 16.27 |
| Expansion | -23485.12 | 4.97 | 15.96 |
| Exponential | -23485.50 | 4.97 | 15.72 |
| Logistic | -23485.37 | 4.97 | 15.83 |

Dataset included strain IS-98, (n=62). Parameters calculated using BEAST 1.6.2. Best model is shown in bold.
BSP = Bayesian Skyline plot, S = strict clock, UCLN = uncorrelated lognormal clock, *tMRCA* = time to most recent common ancestor
